# YAP inactivation in estrogen receptor alpha-positive hepatocellular carcinoma with less aggressive behavior

**DOI:** 10.1038/s12276-021-00639-2

**Published:** 2021-06-18

**Authors:** Youngsic Jeon, Jeong Eun Yoo, Hyungjin Rhee, Young-Joo Kim, Gwang Il Kim, Taek Chung, Sarah Yoon, Boram Shin, Hyun Goo Woo, Young Nyun Park

**Affiliations:** 1 Department of Pathology, Graduate School of Medical Science, Brain Korea 21 Project, Yonsei University College of Medicine, Seoul, Korea; 2 Department of Radiology, Yonsei University College of Medicine, Seoul, Korea; 3 Natural Products Research Center, Korea Institute of Science and Technology, Gangneung, Gangwon-do Korea; 4 Severance Biomedical Science Institute, Yonsei University College of Medicine, Seoul, Korea; 5 Department of Biomedical Systems Informatics, Yonsei University College of Medicine, Seoul, Korea; 6 Department of Physiology, Ajou University School of Medicine, Suwon, Korea; 7 Department of Biomedical Science, Graduate School, Ajou University, Suwon, Korea

**Keywords:** Liver cancer, Gene ontology, Prognostic markers

## Abstract

The expression of estrogen receptor alpha (ERα, encoded by *ESR1*) has been shown to be associated with the prognostic outcomes of patients in various cancers; however, its prognostic and mechanistic significance in hepatocellular carcinoma (HCC) remain unclear. Here, we evaluated the expression of ERα and its association with clinicopathological features in 339 HCC patients. ERα was expressed in 9.4% (32/339) of HCCs and was related to better overall survival (OS; hazard ratio [HR] = 0.11, *p* = 0.009, 95% C.I. = 0.016–0.82) and disease-free survival (DFS, HR = 0.4, *p* = 0.013, 95% C.I. = 0.18–0.85). ERα expression was also associated with features related to more favorable prognosis, such as older age, lower serum alpha-fetoprotein level, and less microvascular invasion (*p* < 0.05). In addition, to obtain mechanistic insights into the role of ERα in HCC progression, we performed integrative transcriptome data analyses, which revealed that yes-associated protein (YAP) pathway was significantly suppressed in *ESR1*-expressing HCCs. By performing cell culture experiments, we validated that ERα expression enhanced YAP phosphorylation, attenuating its nuclear translocation, which in turn suppressed the downstream signaling pathways and cancer cell growth. In conclusion, we suggest that ERα expression is an indicator of more favorable prognosis in HCC and that this effect is mediated by inactivation of YAP signaling. Our results provide new clinical and pathobiological insights into ERα and YAP signaling in HCC.

## Liver cancer: How estrogen receptors hinder tumor growth

Estrogen receptor signaling can act as a brake preventing the progression of an often deadly form of liver cancer. Studies have shown that women are at a lower risk of developing and succumbing to hepatocellular carcinoma (HCC) than men, suggesting a potential role for sex hormones. Researchers in South Korea led by Hyun Goo Woo of the Ajou University School of Medicine, Suwon, and Young Nyun Park of Yonsei University College of Medicine, Seoul, have now shown that expression of the estrogen receptor α is a strong prognostic predictor for HCC. In a survey of patient tumor samples, they found that expression of this hormone receptor is associated with nearly a tenfold increased likelihood of survival. The researchers identified a mechanism by which estrogen receptor α signaling impedes cancerous growth, revealing potential new drug targets.

## Introduction

Hepatocellular carcinoma (HCC) is a highly malignant tumor with a dismal clinical prognosis. The incidence of HCCs is three times higher in males than in females^1^. Moreover, male patients showed worse overall survival (OS) than female patients^2^, implying that sex differences play an important role in HCC progression. Indeed, the sex hormone estrogen and the estrogen receptor (ER) have been found to be associated with the progression of various cancers, including breast cancer, ovarian cancer, and HCC^3,4^.

The two major isoforms of ER are ERα (encoded by *ESR1*) and ERβ (encoded by *ESR2*), and ERα is the most abundant isoform in HCC^5^. Experimental studies have shown that ERα suppresses growth and inflammatory processes in diverse cancer types, including HCC, colorectal cancer, and gastric cancer^6–8^. Consistent with this observation, several studies have suggested that the expression of ERα is associated with better prognosis of HCC patients^9,10^. However, these studies were performed with a limited sample size, requiring further extended evaluation. Moreover, the precise mechanisms by which ERα affects cancer progression are not yet clear.

In this study, to determine the prognostic significance of ERα in HCC patients, we performed an extensive tissue microarray (TMA) analysis of 339 HCC samples, which revealed that ERα expression is an independent indicator of better prognostic outcomes in HCC patients. In addition, the results of our transcriptome data analyses suggested that inactivation of YAP may mediate the tumor-suppressive function of ERα in HCCs. YAP and transcriptional coactivator with PDZ-binding motif (TAZ) are the major downstream effectors of the Hippo pathway, which regulates tissue homeostasis, regeneration, and tumorigenesis^11–13^. Activation of YAP or inhibition of upstream Hippo molecules such as the serine/threonine-protein kinases MST1/2 and large tumor suppressor kinase 1 (LATS1) can lead to nuclear translocation of YAP, which in turn promotes the tumorigenesis and progression of various cancers^14,15^. However, the effect of ERα expression on Hippo/YAP signaling has not yet been fully elucidated. In this study, we demonstrated that ERα expression can attenuate YAP signaling, resulting in suppression of HCC progression. Our results may provide new insights into the clinical and pathobiological significance of ERα and YAP signaling in HCC progression.

## Materials and Methods

### Patients and specimens

Patients who were diagnosed with HCC and underwent curative hepatic resection between May 2000 and February 2011 at Severance Hospital, Yonsei University Medical Center, were enrolled. A total of 339 HCC patients were included in the study, and patients with combined hepatocellular cholangiocarcinoma were excluded. None of the patients had received any preoperative treatment, such as transarterial chemoembolization, percutaneous ethanol injection, radiofrequency ablation, radiation, or systemic chemotherapy. Electronic medical records were reviewed to obtain clinical data, including age, sex, etiology, blood count, and the serum levels of aspartate transaminase, alanine transaminase, albumin, and the tumor marker alpha-fetoprotein (AFP). Histopathologic examination was performed on whole-section hematoxylin-eosin-stained slides prepared from representative formalin-fixed, paraffin-embedded tissue blocks. The following variables were evaluated: tumor size, Edmondson–Steiner grade, tumor multiplicity, tumor capsule formation, presence of microvascular invasion, and fatty change in the tumor.

Patients were routinely followed up by computed tomography or magnetic resonance imaging at intervals of 3–6 months. OS was defined as the time from surgery to death, and DFS was defined as the time from surgery to initial diagnosis of recurrence regardless of location. The mean follow-up time after surgery was 43.0 ± 20.72 months (0–146). The study was approved by the Institutional Review Board of Severance Hospital (IRB No. 4–2018–0748), and the requirement for informed consent was waived.

### Construction of tissue microarrays and immunohistochemistry

TMAs of HCC specimens were constructed using two core biopsies of 2 mm in diameter, which were obtained from paraffin-embedded sections of tissue specimens and arranged into recipient TMA blocks using a trephine apparatus (Superbiochips Laboratories, Seoul, Korea). Immunohistochemistry was performed using antibodies against ERα, EpCAM, K19, CD133, CD24, S100P, and YAP. Brown membranous and/or cytoplasmic staining was considered positive for EpCAM, K19, and CD133; cytoplasmic staining, for CD24; and nuclear staining, for ERα and S100P. Positivity for nuclear and cytoplasmic expression of YAP was concluded if more than 50% of the tumor cells were stained with strong intensity. The details on the antibodies and the experimental conditions are summarized in Supplementary Table S1.

### Transcriptome data analysis

Public data from The Cancer Genome Atlas-Liver Hepatocellular Carcinoma (TCGA-LIHC) dataset were obtained by using the TCGAbiolinks package in R software^16^, and the GSE87630, GSE4024, and GSE14520 datasets were obtained from the NCBI GEO database (https://www.ncbi.nlm.nih.gov/geo/). Integration of the datasets was performed by correcting batch effects using the “Combat” library in R software. Samples with normal tissues were excluded from the datasets.

Gene set analyses were performed using the gProfileR (0.7.0) package in R software and data from the KEGG (http://www.genome.jp/kegg/) and REAC (http://www.reactome.org/) databases. Coordinated gene regulation was identified using GSEA (https://www.gsea-msigdb.org/gsea, version 3.0). Gene sets from previous studies, including the gene sets for stemness/stem cell genes^17^ and the prognostic HCC classifiers of Hoshida’s classification^18^ and Boyault’s classification^19^. Prediction of the HCC groups by the prognostic classifiers was performed with the nearest template prediction (NTP) algorithm with a false discovery rate (FDR) <0.05 as the criterion for statistical significance as described previously^20^. Detailed information on the datasets is provided in Supplementary Table S2.

The oncoactivity score was calculated based on the differential enrichment of oncogene activation tumor suppressor gene (TSG) repression as follows: Oncoactivity = ES_oncogene_ − ES_TSG_, where ES was the calculated enrichment score of the indicated genes. The lists of oncogenes (*n* = 674)^21^ and TSGs (*n* = 1088)^22^ were obtained from previous studies.

### Cell culture

The human HCC cell lines HepG2 and Hep3B were purchased from the American Type Culture Collection (ATCC, Manassas, VA, USA) and grown in minimum essential medium (MEM, Gibco, Carlsbad, MD, USA), Dulbecco’s modified Eagle’s medium (DMEM, Gibco), and Roswell Park Memorial Institute 1640 medium (RPMI 1640, Gibco) supplemented with 10% fetal bovine serum (Gibco), 100 U/mL penicillin, and 100 μg/mL streptomycin at 37 °C in a humidified atmosphere with 5% CO_2_.

### Cloning and establishment of overexpression cell lines

For the cloning of tagged *ESR1* coding gene sequences into pCDH-CMV-EF1-puro, the *ESR1* gene was amplified using total RNA extracted from normal liver tissue. PCR was performed using specific primers containing a 5′-extension and NotI (NEB, Ipswich, MA, USA) and XbaI (NEB) restriction sites with CloneAmp HiFi PCR Premix (Thermo Fisher Scientific, San Jose, CA, USA). Subsequently, the amplicons were digested with NotI and XbaI and cloned into the pCDH-CMV-EF1-puro vector using an In-Fusion^®^ cloning system according to the manufacturer’s recommendations. The details of the primer sequences and thermal cycling conditions are summarized in Supplementary Table S3.

HepG2 and Hep3B cells were transfected with pCDH-CMV-EF1-puro containing the tagged *ESR1* coding sequence along with the gag-pol and VSV-G plasmids (plasmids 14887 and 8454, respectively; Addgene, Cambridge, MA, USA) using Lipofectamine^®^ 3000 (Invitrogen, Carlsbad, CA, USA) according to the manufacturer’s recommendations. After transfection, stable *ESR1*-expressing cells were selected with 0.5–1.0 µg/mL puromycin (Sigma-Aldrich Co., St. Louis, MO, USA) for 4 weeks. To confirm the specificity and efficiency of *ESR1* overexpression, we analyzed mRNA and protein expression using qRT-PCR and Western blotting.

### Immunofluorescence analysis

Hep3B cells were seeded on microscope cover glasses in 12-well plates. After 24 h, the cells were fixed with 4% paraformaldehyde for 30 min. After permeabilization with PBS containing 0.2% Tween 20 for 30 min at 4 °C, the cells were blocked with freshly prepared 2% bovine serum albumin (BSA) for 1 h. The fixed cells were stained with an anti-YAP (1:400, sc-271134, Santa Cruz) antibody in 2% BSA for an hour at room temperature and then with Alexa Fluor^®^ 488-conjugated goat anti-mouse IgG as the secondary antibody for 1 h at room temperature. Finally, the cells were washed with cold PBS, and the cover glasses were mounted with DAKO^®^ Fluorescent Mounting Medium (DakoCytomation, Carpinteria, CA, USA). The cells were examined under a TCS SP5 confocal microscope system (Leica, Deerfield, IL, USA).

### Dual-luciferase reporter assay

Cells were cotransfected with 100 ng of each reporter construct and 0.25 ng of the pNL1.1.TK vector (Promega, Madison, WI, USA) per well in white-bottom 96-well plates (SPL Life Science, Pocheon, Korea) using 0.5 µl of FuGENE^®^ HD Transfection Reagent (Promega). To determine the effect of YAP/TEAD promoter activity, cells in each well were cotransfected with reporter constructs such as pGL3.0-basic (Promega) and 8xGITTC-luciferase (Addgene, plasmid #34615, Cambridge, MA, USA) and the pNL1.1.TK vector. Forty-eight hours post transfection, luciferase activity was measured with a Nano-Glo^®^ Dual Luciferase^®^ Reporter Assay System (Promega) according to the manufacturer’s recommendations. Relative firefly luciferase activity was normalized to NanoLuc^™^ luciferase activity to adjust for variations in the transfection efficiency.

### Western blotting and quantitative real-time PCR analysis

The primary antibodies used were rabbit anti-ERα (8644, Cell Signaling Technology [CST], Danvers, MA, USA), rabbit anti-phospho-YAP/S109 (13008, CST), rabbit anti-phospho-YAP/S127 (4911, CST), rabbit anti-YAP (14074, CST), rabbit anti-phospho-LATS/S1079 (8654, CST), rabbit anti-LATS1 (3477, CST), rabbit anti-phospho-MST1/2/T183/180 (3681, CST), rabbit anti-MST1 (3681, CST), rabbit anti-GAPDH (2118, CST), and rabbit anti-Histone H3 (cs-10809, Santa Cruz, California, CA, USA). Horseradish peroxidase-conjugated anti-rabbit IgG (7047, CST) and anti-mouse IgG (7076, CST) were used as secondary antibodies (for details, see Supplementary Table S1).

Quantitative real-time PCR was performed using iQTM SYBR Supermix (Bio-Rad, Hercules, CA, USA). The details of the primer sequences and thermal cycling conditions are summarized in Supplementary Table S3.

### siRNA-mediated knockdown experiments

*ESR1* siRNA (MISSION^®^ select predesigned siRNAs; SASI_Hs01_00078594, Sigma-Aldrich Co.) and MISSION^®^ siRNA Universal Negative Control (Sigma-Aldrich Co.) were transfected into established ERα (−) and ERα (+) stable cells using Lipofectamine^®^ RNAiMAX Transfection Reagent (Invitrogen) according to the manufacturer’s recommendations. For the dual-luciferase reporter assay, 100 ng of each construct, the pNL1.1.TK vector (0.25 ng) and *ESR1* siRNA (50 nM) or siRNA Universal Negative Control (50 nM) per well were cotransfected in 96-well white-bottom plates using Lipofectamine^®^ 3000 Reagent (0.7 µl, Invitrogen) according to the manufacturer’s recommendation.

### Statistical analysis

Statistical analysis was conducted using SPSS (version 23.0.1; SPSS Inc., Chicago, IL, USA) or R software (version 3.4.0; Vienna, Austria).

## Results

### ERα expression is associated with clinical and pathological features of HCC

**Fig. 1:**
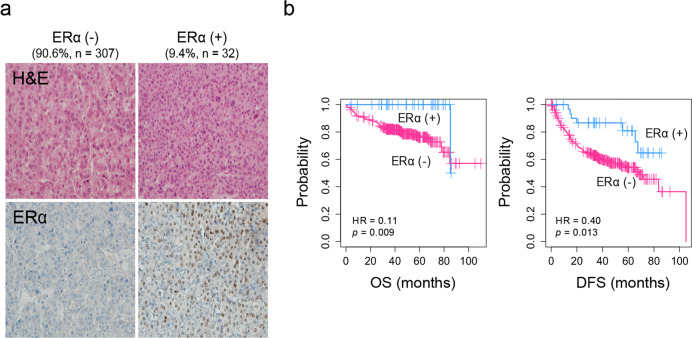
ERα expression is associated with less aggressive clinical pathological features of HCC. **a** Histopathological images of ERα (−) and ERα (+) HCC tissue. ERα is expressed in the nuclei of tumor cells. **b** Kaplan–Meier curves of overall survival and disease-free survival for patients with ERα (+) (*n* = 32) and ERα (−) HCC (*n* = 307).

ERα expression was evaluated using immunohistochemical staining of a TMA of samples from 339 HCC patients. We observed that 9.4% (32/339) of the HCCs expressed ERα (Fig. 1a). Clinicopathological features were evaluated according to the expression of ERα, which revealed that compared to ERα (−) HCCs, ERα (+) HCCs, were significantly associated with older age (>60 years, *p* < 0.05, Student’s T-test), lower serum AFP levels (3.6 IU/mL, *p* < 0.001, Student’s T-test), a lower incidence of tumor microvascular invasion (38%, 12/32, *p* = 0.029, Fisher’s exact test), and a higher incidence of fatty change in the tumor (46.9%, 15/32, *p* = 0.017; Table 1). Kaplan–Meier survival analysis revealed that the patients with ERα (+) HCC had better prognostic outcomes of OS (HR = 0.11, *p* = 0.009, Fisher’s exact test) and DFS (HR = 0.40, *p* = 0.013, Fig. 1b). Univariate and multivariate Cox regression analyses also confirmed that ERα expression is an independent predictor of the prognostic outcomes of HCC patients (*p* < 0.05, one-way *Chi*-square test Table 2).

**Table 1:** Clinicopathological characteristics of patients with HCC according to the ERα protein expression level.

Clinicopathological feature	ERα protein-positive HCCs	ERα protein-negative HCCs	*p* value
	*n* = 32 (9.4%)	*n* = 307 (90.6%)	
Age (years; median, IQR)	60 (51–68)	55 (48–63)	0.015
Sex (male/female, %)	26 (81%)/6 (19%)	252 (83%)/53 (17%)	1
Etiology (HBV/HCV/Alcohol/Unknown, %)	25 (78%)/1 (3%)/1 (3%)/5 (16%)	252 (82%)/17 (6%)/14 (5%)/24 (8%)	0.462
Serum AST (IU/L; median, IQR)	28 (22–41)	30 (23–40)	0.934
Serum ALT (IU/L; median, IQR)	31 (20–49)	31 (22–45)	0.852
Serum albumin (g/dL; median, IQR)	4.3 (4.1–4.6)	4.4 (4.1–4.7)	0.61
Serum platelets (×1000/μL; median, IQR)	174 (119–201)	162 (133–210)	0.343
Serum alpha-fetoprotein (IU/mL; median, IQR)^a^	3.6 (2.5–27.1)	24.9 (4.6–279.5)	<0.001
Serum PIVKA-II (AU/mL; median, IQR)^b^	58.0 (24.0–346.0)	81.0 (33.5–539.0)	0.189
TNM stage (stage I/II/III, %)	17 (53%)/14 (44%)/1 (3%)	105 (34%)/186 (61%)/16 (5%)	0.104
HCC pathology			
Differentiation (Edmonson–Steiner Grade I/II/III, %)	5 (16%)/22 (69%)/5 (16%)	21 (7%)/224 (73%)/62 (20%)	0.193
Diameter of the largest tumor (cm; median, IQR)	2.7 (1.8–4.3)	3.2 (2.2–4.5)	0.782
Tumor multiplicity	3 (9%)	41 (13%)	0.075
Tumor capsule formation (absent/partial/complete, %)	5 (16%)/14 (44%)/13 (40%)	54 (18%)/168 (55%)/85 (28%)	0.302
Microvascular invasion (%)	12 (38%)	182 (59%)	0.029
Tumor fatty change (absent/present, %)	17 (53.1%)/15 (46.9%)	223 (73.1%)/82 (26.9%)	0.017
Nontumor liver pathology			
Chronic hepatitis or cirrhosis (%)	30 (93.8%)	294 (95.8%)	0.642

IQR interquartile range, SD standard deviation.

^a^Serum alpha-fetoprotein was not available for one patient.

^b^Serum PIVKA-II was not available for 11 patients.

**Table 2:** Univariate and multivariate Cox regression analysis of overall survival of HCC patients.

Variable		Univariate analysis			Multivariate analysis		
	No. of patients (*n* = 339)	HR	95% C.I.	*p* value	HR	95% C.I.	*p* value
Age (years)		1.3	0.78–2.1	0.33			
≤60	224						
>60	115						
Sex		1.4	0.74–2.7	0.3			
Male	277						
Female	62						
Serum ALT (IU/L)		1.2	0.65–2	0.62			
≤50	274						
>50	65						
Serum AST (IU/L)		1.8	0.95–3.2	0.07			
≤50	299						
>50	40						
Alpha-fetoprotein		1.6	0.85–2.9	0.15			
≤1000	294						
>1000	44						
Albumin		0.9	0.5–1.6	0.32			
≤4.5	219						
>4.5	120						
Cirrhosis		1.4	0.84–2.2	0.22			
Absent	150						
Present	189						
Tumor size		1.6	0.98–2.5	0.62			
<5 cm	264						
≥5 cm	75						
Tumor multiplicity		1.5	0.76–2.9	0.25			
Absent	302						
Present	37						
Microvascular invasion		1.7	1–2.7	0.05	1.12	0.34–2.28	0.81
Absent	145						
Present	194						
Differentiation		1.9	1.1–3.2	0.027	1.76	0.64–4.79	0.27
I	122						
II-III	217						
ERα protein expression		0.24	0.07–0.8	0.031	0.25	0.08–0.83	0.038
Negative	307						
Positive	32						

HR hazard ratio, CI confidence interval.

### Identification of the molecular traits associated with *ESR1* expression in HCC

**Fig. 2:**
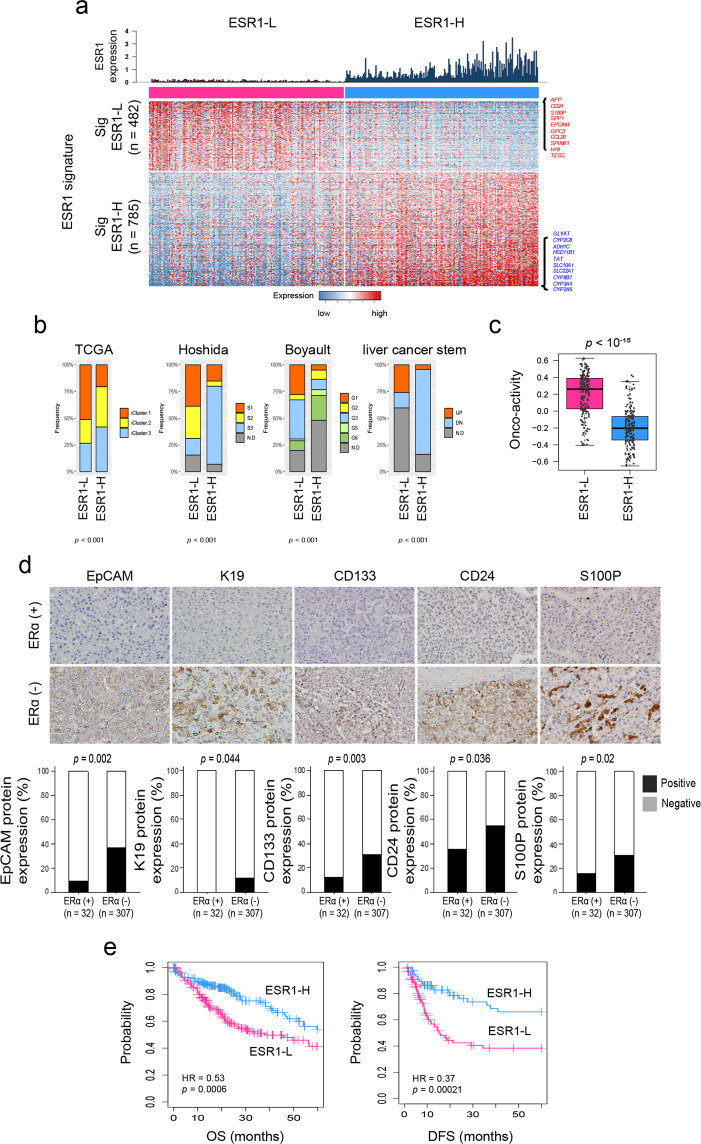
Transcriptomic traits of HCC associated with the expression status of *ESR1*. **a** Heatmap showing the expression pattern of DEGs (Sig ESR1-L, *n* = 482; Sig ESR1-H, *n* = 785). The top ten DEGs are indicated on the right side. **b** Boxplots showing the frequencies of the subtypes predicted by the previous HCC classifiers (i.e., TCGA, Hoshida’s, Boyault’s, and liver cancer stem cell features). **c** Boxplot showing the oncoactivity scores among the HCC subtypes. **d** Images of immunohistochemical staining for EpCAM, K19, CD133, CD24, and S100P in the HCC TMA. Bar charts showing the differences in the expression of EpCAM, K19, CD133, CD24, and S100P between ERα (+) and ERα (−) HCCs. **e** Kaplan–Meier curves of OS and DFS for patients in the ESR1-H (*n* = 185) and ESR1-L (*n* = 186) groups in TCGA-LIHC.

To evaluate the molecular characteristics associated with ERα expression, we analyzed the HCC transcriptome data of the TCGA-LIHC dataset (*n* = 371)^16^. The HCC patients were stratified according to the expression level of *ESR1* mRNA into the *ESR1*-high (ESR1-H, *n* = 185) and *ESR1*-low (ESR1-L, *n* = 186) groups, with the median value of the *ESR1* level across the samples considered the cutoff value. Unsupervised clustering analysis using variably expressed genes (median absolute deviation [MAD] >0.5, *n* = 7094) revealed that the transcriptome of the HCC patients was readily classifiable based on the *ESR1* expression status. Specifically, 155 of 185 ESR1-H HCCs (83.8%) and 148 of 186 ESR1-L HCCs (79.6%) were clustered together (Supplementary Fig. S1), which may indicate that the molecular traits of ERα expression are well reflected in the HCC transcriptome. Next, we identified the differentially expressed genes (DEGs) between the ESR1-H and ESR1-L HCC groups as the ESR1 signatures “Sig ESR1-L” (*n* = 482) and “Sig ESR1-H” (*n* = 785) (*p* < 10^–6^, permutation *t*-test with fold change [FC] >0.5; Supplementary Table S4). Sig ESR1-H was enriched with metabolism-related genes, including *CYP2A6* (FC = 3.83), *CYP3A4* (FC = 3.78), *CYP8B1* (FC = 3.38), and *CYP1A2* (FC = 2.33) (Fig. 2a and Supplementary Fig. S2), whereas Sig ESR1-L exhibited enrichment with stemness-related or tumor aggressiveness-related genes, such as *AFP* (FC = −2.70), *CD24* (FC = −2.27), *S100P* (FC = −2.01), *SPP1* (FC = −1.89), *EPCAM* (FC = −1. 83), and *KRT19* (FC = −1.01). Moreover, we found that compared to the ESR1-H group, the -ESR1-L group exhibited higher expression of HALLMARK oncogenic features such as “MYC_TARGETS_V1” (*n* = 200), “E2F_TARGET” (*n* = 200), “G2M_CHECKPOINT” (*n* = 200), “MYC_TARGETS_V2” (*n* = 58), and “UNFOLDED_PROTEIN_RESPONSE” (*n* = 113) (Supplementary Fig. S3). In addition, we evaluated the expression of the previously known prognostic classifiers of HCC (i.e., TCGA subtype^23^, Hoshida’s classification^18^, Boyault’s classification^19^, and liver cancer stem cell features^17^) in the TCGA-LIHC dataset by applying the NTP algorithm (for details, see Materials and Methods). The ESR1-L group exhibited enrichment of the aggressive prognostic classifiers such as iCluster1 in TCGA subtype, S1 and S2 in Hoshida’s classification, G1 and G3 in Boyault’s classification, and UP in liver cancer stem cell features (*p* < 0.001, chi-square test); in contrast, the ESR1-H group was enriched in the less aggressive prognostic classifiers, such as S3 in Hoshida’s classification, G5 and G6 in Boyault’s classification, and DOWN in liver cancer stem cell features (*p* < 0.001, chi-square test, Fig. 2b). When we calculated the oncoactivity score for each sample based on the differentially upregulated expression of the oncogenes and counterbalanced suppression of the TSGs (i.e., ES_oncogene_ − ES_TSG_) as described previously^24^ (for details, see Materials and Methods), we observed significantly higher oncoactivity scores in ESR1-L patients than in ESR1-H patients (*p* < 10^−15^, Fig. 2c). We also found that ESR1-L patients expressed stemness- and tumor aggressiveness-related genes (*CD24*, *S100P, EPCAM*, and *KRT19*), which have been shown to play critical roles in the development and progression of tumors^25,26^. This finding was validated by immunohistochemical analysis of the 339 HCC specimens, demonstrating the differential expression of EpCAM, K19, CD133, CD24, and S100P between the ERα (+) and ERα (−) HCCs (*p* < 0.05, Fisher’s exact test; Fig. 2d). Correspondingly, Kaplan–Meier survival analysis revealed that the ESR1-H patients exhibited better OS (HR = 0.53, *p* < 10^−3^) and DFS (HR = 0.37, *p* < 10^−3^) than the ESR1-L patients (Fig. 2e). ESR1-H status was also significantly associated with older age (>62 years, *p* < 0.001) and a lower serum AFP level (*p* < 0.001) (Supplementary Table S5). Univariate and multivariate analyses confirmed that *ESR1* expression was an independent prognostic predictor of the clinical outcomes of patients in TCGA-LIHC (*p* < 0.05, Supplementary Table S6). These results may strongly indicate that ERα (or ESR1) expression is a good indicator of better prognostic outcomes of HCC and is associated with alterations in the transcriptomic traits of HCC.

**Fig. 3:**
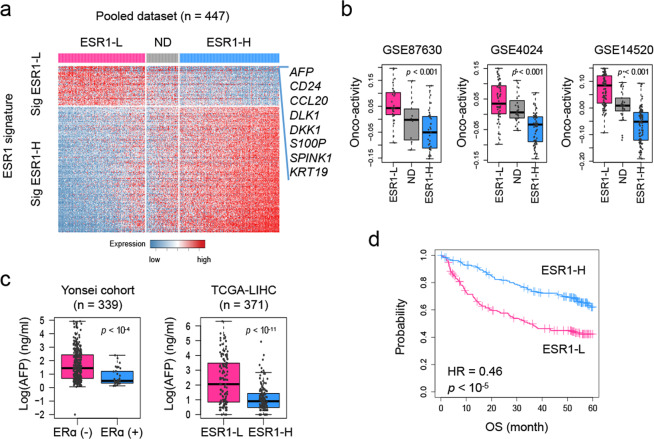
Validation of the functional and clinical significance of *ESR1* expression in a pooled HCC dataset. **a** Heatmap showing the shared differentially upregulated (ESR1-L; *n* = 63) and downregulated (ESR1-H; *n* = 199) genes in a pooled dataset including GSE87630, GSE4024, and GSE14520. The DEGs in the pooled dataset were defined as genes with a fold change >1 and were assessed using Student’s *t*-test (*p* < 0.01) in the comparison of ESR1-H with ESR1-L. **b** Boxplots showing the oncoactivity score (the enrichment score for the transition from an oncogene to a TSG signature) of each group. **c** Plots showing the serum levels of AFP in the Yonsei cohort and TCGA-LIHC. **d** Kaplan–Meier OS curve for patients with ESR1-H and ESR1-L HCCs.

Next, we further validated the class predictability of the ESR1 signature by constructing a pooled dataset of HCC transcriptomes (*n* = 447), including data from GSE87630 (*n* = 64), GSE4024 (*n* = 139), and GSE14520 (*n* = 244). By applying the NTP algorithm, we stratified the dataset into three groups: ESR1-H (*n* = 201), ESR1-L (*n* = 177), and N.D. (*n* = 69, not determined, FDR ≥.05; Fig. 3a). We demonstrated the higher oncoactivity scores in the ESR1-L group compared to the ESR1-L group in each of the datasets (*p* < 0.001, Fig. 3b; for details, see Material and Methods). In addition, we identified the DEGs between the ESR1-H and ESR1-L groups (*p* < 0.01, permutation *t*-test and FC >1.0), revealing that *AFP* expression had the highest FC between the groups (FC = 3.34, *p* < 10^−12^). In support of this finding, we observed that HCC samples from patients with higher serum levels of AFP (>300 ng/mL) were more frequently ERα (−) tumors than ERα (+) tumors in our data and were more frequently ESR1-L tumors than ESR1-H tumors in TCGA-LIHC data (*p* < 0.001, chi-square test; Fig. 3c). Kaplan–Meier survival analysis also successfully demonstrated the prognostic significance of *ESR1* expression in the pooled dataset (*n* = 378, HR = 0.46, *p* < 10^−5^; Fig. 3d). Considering these results collectively, we suggest that *ESR1* expression plays a pivotal role in transcriptomic alterations in association with the heterogeneous progression of HCC.

### ERα expression inhibits HCC progression by inactivating the YAP pathway

**Fig. 4:**
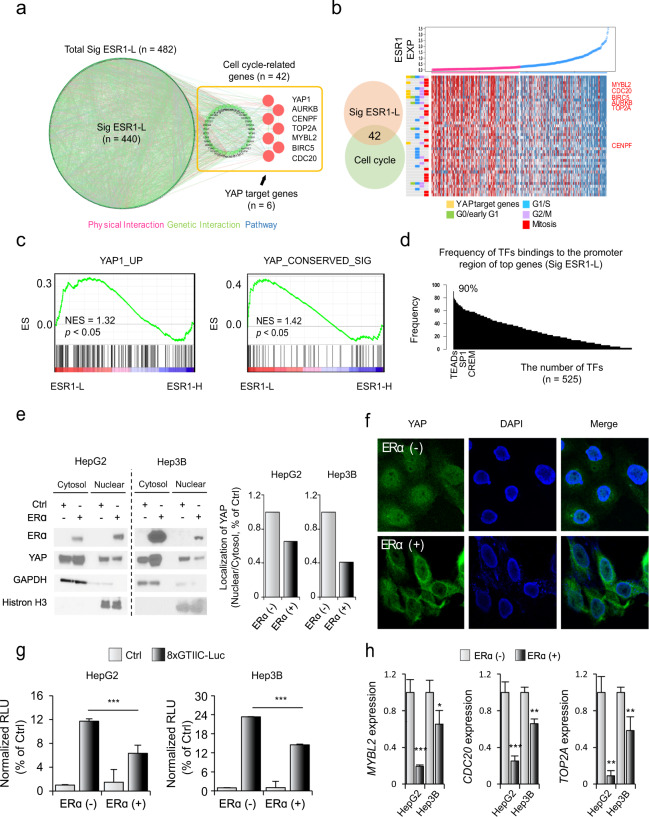
YAP may mediate the aggressive phenotype of ESR1-L HCC. **a** A genetic network of Sig ESR1-L genes was constructed showing physical interactions (pink), genetic interactions (green), and pathways (blue) by using GeneMANIA software in Cytoscape (version 3.4.1). Cell cycle-related genes and YAP target genes in Sig ESR1-L are indicated. **b** Plot showing the gradually increase in ESR1 expression (top). Heatmap showing the expression of YAP target genes and cell cycle-related genes in Sig ESR1-L (bottom). Cell cycle groups, such as G0/early G1-, G1/S-, G2/M-, and mitosis-related groups, are indicated. **c** GSEA results showing the enrichment of YAP activation-related signatures (e.g., YAP1_UP and YAP_CONSERVED_SIG). **d** Bar plots showing the frequency of transcription factors binding to the promoters of the top-ranked genes in Sig ESR1-L (*n* = 50). This database was investigated using GeneHancer (https://www.genecards.org/). **e** The amount of YAP protein that translocated from the cytosol into the nucleus was assessed by Western blotting after nucleocytoplasmic fractionation. GAPDH and histone H3 were used as nuclear and cytoplasmic control markers, respectively (left). The amount of YAP protein that was translocated from the cytosol into the nucleus was assessed by Western blotting after nucleocytoplasmic fractionation using ImageJ (1.8.0_172) (right). **f** Confocal immunofluorescence of YAP and 4′,6-diamidino-2-phenylindole (DAPI). DAPI (blue) and YAP (green) were detected by confocal microscopy as described in the Materials and Methods. **g** Dual-luciferase assays showed that the enhanced normalized relative luminescence unit (RLU) value of the 8xGTIIC construct was decreased in ERα-expressing HCC cell lines. The pGL3.0-basic vector was used as a control (Ctrl). Statistical significance is indicated (Ctrl vs. ERα; *p < 0.05, **p < 0.01, and ***p < 0.001, Student’s *t*-test). **h** Bar plots showing the expression of YAP target genes (e.g., *MYBL2*, *CDC20*, and *TOP2A*) in ERα (+) and ERα (−) liver cancer cell lines (HepG2 and Hep3B). The expression levels of each gene were normalized to the expression level of *ACTB*. The data were presented as the mean ± SD values (***p < 0.001, Student’s *t*-test).

We next sought to identify the underlying key downstream molecules that potentially drive the aggressive phenotype of ESR1-L. We constructed a genetic network of Sig ESR1-L by using the KEGG and REAC databases (for details, see Materials and Methods) and found that the gene set of cell cycle-related genes (9.95%, 42/482) was the most significantly enriched (Fig. 4a and Supplementary Fig. S2). We found that among the cell cycle-related genes, YAP-related target genes (i.e., *MYBL2*, *CDC20*, *BIRC5*, *AURKB*, *TOP2A*, and *CENPF*) and G2/M-related genes were more highly differentially expressed in ESR1-L than in ESR1-H (Fig. 4b). Gene set enrichment analysis also revealed enriched expression of “YAP1_UP”^27^ and “YAP_CONSERVED_SIG”^28^ genes in ESR1-L (*p* < 0.05, Fig. 4c). Indeed, YAP binding to B-MYB (encoded by the *MYBL2* gene) is known to induce the expression of G2/M genes^29^. Moreover, we found that the promoter regions of the Sig ESR1-L genes frequently contained the predicted transcription factor (TF) binding sites for TEA domain family members (TEADs) (Fig. 4d), which are known to be activated by YAP^12^. Thus, it is plausible that *ESR1* expression may inactivate the YAP pathway, resulting in suppression of cell cycle-related genes and tumor progression.

To verify whether *ESR1* expression can affect the YAP pathway, we established an ERα overexpression system in liver cancer cell lines (HepG2 and Hep3B) that have lower expression levels of *ESR1* (Supplementary Fig. S4a). We demonstrated the tumor-suppressive effect of ERα (+) cells compared to ERα (−) cells (*p* < 0.001, Supplementary Fig. S4b). In addition, we evaluated whether ERα expression can regulate YAP activity by examining the cytosolic and nuclear levels of YAP using Western blot and immunofluorescence analyses. We demonstrated that compared to ERα (−) cells, ERα (+) cells significantly suppressed the nuclear translocation of YAP (Fig. 4e,f). Furthermore, by performing YAP/TAZ reporter assays, we demonstrated that compared to ERα (−) cells, ERα (+) cells markedly suppressed YAP transactivation (all *p* < 0.001, Fig. 4g). Additionally, we observed that the expression of TEAD target genes (i.e., *MYBL2*, *CDC20*, and *TOP2A*) was suppressed in ERα (+) cells compared with ERα (−) cells (Fig. 4h). These results suggest that ERα expression leads to inactivation of YAP and suppression of its downstream target genes.

### ERα-induced YAP inactivation may be mediated by Hippo activation

**Fig. 5:**
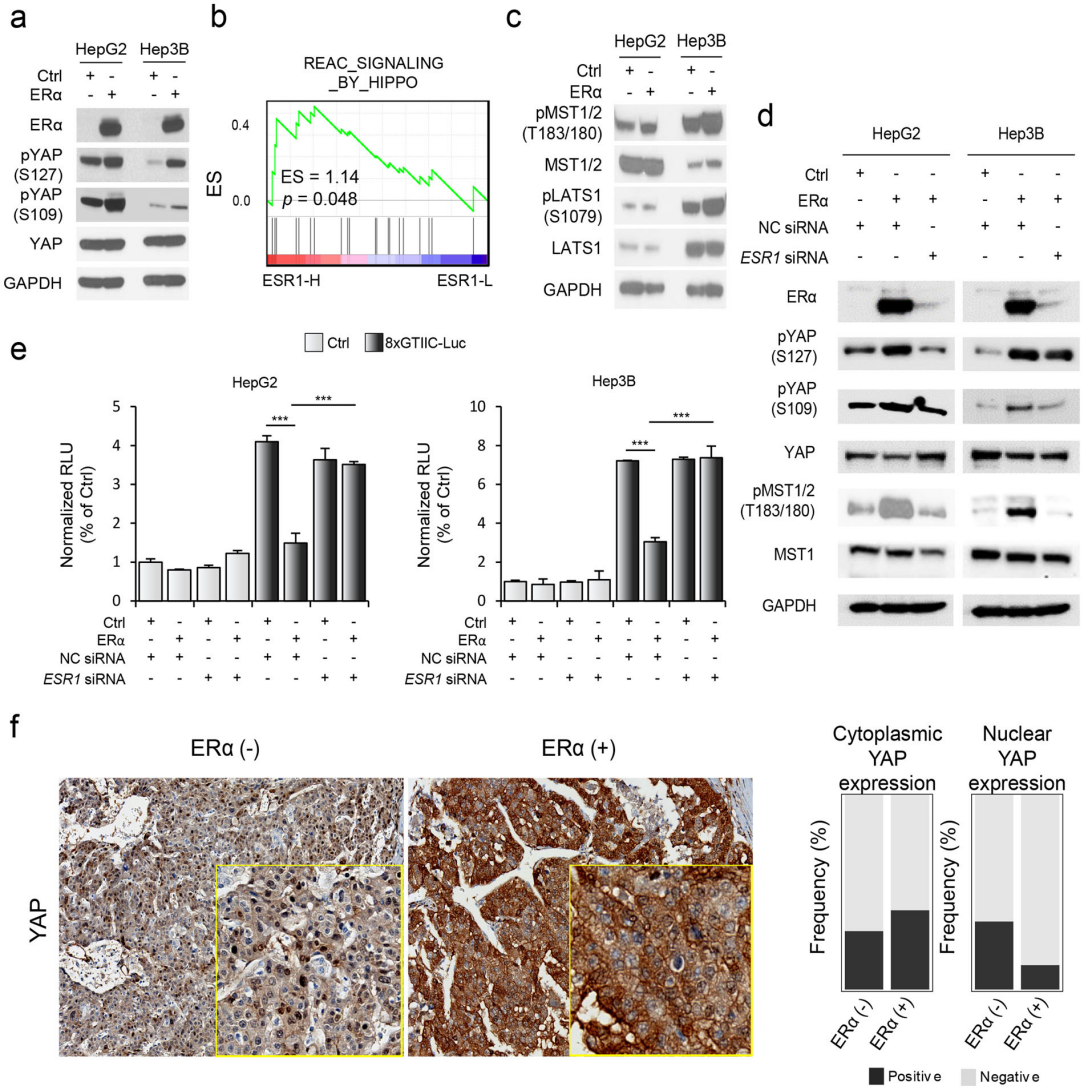
ERα expression attenuates the translocation of YAP via phosphorylation of MST1/2. **a** Expression of YAP and phosphorylation of YAP at Ser109 and Ser127 were shown by Western blot analysis. **b** GSEA results showing the enriched expression of Hippo pathway-related genes. NES normalized enrichment score. **c** Levels of phospho-MST1/2 (T183/180), MST1, phospho-LATS1 (S1079), and LATS1 were shown by Western blot analysis. **d** ESR1 siRNA (50 nM) was used to treat ERα (+) HCC cell lines, and the phosphorylation of YAP (Ser127 and S109) and MST1/2 (T183/180) was shown by Western blot analysis. **e** Bar plots showing the normalized RLU values of the 8xGTIIC construct in the absence of ERα resulting from transfection of ESR1 siRNA (50 nM) in HepG2 (left) and Hep3B (right) liver cancer cells. The pGL3.0-basic vector (Ctrl) and NC (nontarget control) siRNA were used as controls. Statistical significance is indicated (*p < 0.05, **p < 0.01, and ***p < 0.001, Student’s *t*-test). **f** Immunohistochemical analysis of YAP showing its nuclear and cytoplasmic expression in ERα (−) and ERα (+) HCCs (left). Boxplots showing significant differences in nuclear and cytoplasmic expression of the YAP protein between ERα (−) and ERα (+) HCCs (*p* < 0.05, Fisher’s exact test; right).

Next, to identify the mechanisms underlying YAP inactivation by ERα, we examined the YAP phosphorylation status. Compared to ERα (−) cells, ERα (+) cells exhibited enhanced phosphorylation of YAP at Ser109 and Ser127 (Fig. 5a). As these sites were previously found to be phosphorylated by LATS1 and MAST1/2, core regulatory kinase proteins in the Hippo pathway^30,31^, we further evaluated whether Hippo signaling is also involved in ERα-mediated YAP inactivation. We demonstrated that Hippo pathway genes (Reactome_signaling_by_hippo, *n* = 20) were prominently expressed in the ESR1-H group compared to the ESR1-L group in the TCGA-LIHC dataset (*p* = 0.048, NES = 1.14, Fig. 5b). In support of this finding, phosphorylation of the upstream Hippo signaling molecules LATS1 and MST1/2 was increased in ERα (+) cells compared with ERα (−) cells (Fig. 5c)^12,32^. These results suggest that ERα-induced YAP inactivation is mediated at least in part through activation of Hippo signaling.

To further verify the effect of ERα on YAP, we performed small interfering RNA (siRNA)-mediated *ESR1* knockdown experiments. Transfection of siRNAs targeting ESR1 suppressed ERα-induced phosphorylation of YAP and MST1/2 (Fig. 5d). In addition, we evaluated YAP/TAZ promoter activities by cotransfecting the YAP/TAZ reporter construct with *ESR1* siRNA, and the results revealed that *ESR1* knockdown enhanced the suppressive effect of ERα on YAP/TAZ promoter activity (Fig. 5e). Furthermore, we validated our findings by immunohistochemical staining analysis of YAP in HCC tissues. ERα (−) HCC specimens showed increased nuclear expression of YAP (34.9%, *p* = 0.01) compared with that in ERα (+) HCC specimens (12.5%), although the cytoplasmic expression levels of YAP were not different between the groups (Fig. 5f). Considering these results collectively, we suggest that ERα enhances the phosphorylation of Hippo/YAP signaling molecules and attenuates downstream signaling in this pathway, which in turn results in suppression of HCC growth.

## Discussion

In this study, we demonstrated that ERα expression is an independent predictor of better prognostic outcomes of HCC patients. In addition, by performing transcriptome data analyses, immunohistochemistry, and cell culture experiments, we demonstrated that the YAP pathway is involved in ERα-mediated suppression of HCC growth.

Previously, numerous studies have shown that YAP/TAZ function as oncogenes in many cancers^33^. Activated YAP can sustain positive feedback for the expression of cell cycle-related genes to promote tumorigenesis^34,35^. Moreover, YAP has been shown to induce the expression of stemness-related genes, promoting an aggressive tumor phenotype^33^. Indeed, YAP can reprogram nonstem tumor cells into cells with cancer stem cell attributes^36^. Correspondingly, the association of YAP with less favorable prognostic outcomes has been reported in HCC^37,38^. However, contradictory evidence for the tumor-suppressive functions of YAP/TAZ has also been reported^39^. A recent study showed that YAP/TAZ exert a tumor-promoting function but that activation of YAP/TAZ in peritumoral hepatocytes can suppress primary liver tumor progression^40^. This pattern may indicate that YAP/TAZ exhibit both tumor-suppressive and tumor-promoting activity depending on their expression in tissues.

We demonstrated that ERα inactivates YAP signaling, resulting in suppression of HCC growth. A recent study demonstrated that the YAP1 and TEAD4 proteins act as coregulators of ERα on enhancers^41^. Binding of YAP1/TEAD4 to ERα-bound enhancers is required for the induction of ERα target genes and tumor growth. However, the effect of ERα on the YAP pathway has not been clearly shown. We demonstrated that the expression of ERα triggers the phosphorylation of Hippo kinases (LATS1 and MST1) and YAP, which attenuates the nuclear translocation of YAP.

It should be noted that we did not evaluate how ERα phosphorylates Hippo/YAP proteins, requiring further extended studies in the near future. In addition, many of the multifaceted interactions of the Hippo/YAP pathways have been shown previously, including those that induce changes in mechanotransduction, inflammation, and oncogenic signaling^33^, which should be evaluated to understand more relevant mechanisms of ERα expression in HCC progression.

In conclusion, we suggest that the expression of ERα is an independent predictor of more favorable prognostic outcomes in HCC patients. Inactivation of YAP by ERα may contribute to the acquisition of a less aggressive phenotype by HCC tumors. Thus, targeting YAP could be a promising therapeutic strategy, especially for patients with ERα (−) HCC.

## Supplementary information

supplementary data
