# Supplementary material for: YAP inactivation in estrogen receptor alpha-positive hepatocellular carcinoma with less aggressive behavior
Source: Exp Mol Med. 2021 Jun 18;53(6):1055–67. doi: 10.1038/s12276-021-00639-2 (PMC8257598; doi:10.1038/s12276-021-00639-2)
Supplement: Supplementary file 1 — supplementary data [file 12276_2021_639_MOESM1_ESM.docx]

**Supplementary Information for:**

**YAP inactivation in estrogen receptor alpha positive hepatocellular carcinoma with less aggressive behavior**

Youngsic Jeon^1^, Jeong Eun Yoo^1^, Hyungjin Rhee^2^, Young-Joo Kim^3^, Gwang Il Kim^4^, Taek Chung^5^, Sarah Yoon^6,7^, Boram shin^6,7^, Hyun Goo Woo^6,7^, Young Nyun Park^1^

^1^Department of Pathology, Graduate School of Medical Science, Brain Korea 21 Project, Yonsei University College of Medicine, Seoul, Korea

^2^Department of Radiology, Yonsei University College of Medicine, Seoul, Korea

^3^Natural Products Research Center, Korea Institute of Science and Technology, Gangneung, Gangwon-do, Korea

^4^Severance Biomedical Science Institute, Yonsei University College of Medicine, Seoul, Korea

^5^Department of Biomedical Systems Informatics, Yonsei University College of Medicine, Seoul, Korea

^6^Department of Physiology, Ajou University School of Medicine, Suwon, Korea

^7^Department of Biomedical Science, Graduate School, Ajou University, Suwon, Korea

# Supplementary Tables

## Supplementary Table 1. List of antibodies used in immunohistochemistry and Western blotting

| Antibody | Source | Dilution (IHC) | Dilution (WB/IF) | Antigen retrieval |
| --- | --- | --- | --- | --- |
| ERα (mouse mAb;SP1) | Ventana Medical Systems (AZ, USA) | prediluted |  | Automated immunostainer |
| EpCAM (mouse mAb; VU-1D) | Calbiochem (Darmstadt, Germany) | 1:3000 | - | Microwave, Citrate (pH 6.0) |
| K19 (mouse mAb; RCK108) | DAKO (Glostrup, Denmark) | 1:100 | - | Enzyme, Protease K |
| CD133(mouse mAb; W6B3C1) | Miltenyi Biotec (Teterow, Germany) | 1:25 | - | Microwave, Citrate (pH 6.0) |
| CD24 (goat pAb) | Santa cruz (CA,USA) | 1:100 | - | Microwave, Citrate (pH 6.0) |
| S100P (mouse mAb; 16/S100P) | BD Transduction Laboratories (CA, USA) | 1:100 | - | Microwave, Citrate (pH 6.0) |
| YAP (rabbit mAb; D8H1X) | Cell Signaling Technology (MA, USA) | 1:100 | 1:1000/1:500 | Automated immunostainer |
| ERα (rabbit mAb; D8H8) | Cell Signaling Technology (MA, USA) |  | 1:1000 |  |
| p-YAP (S109, rabbit mAb) | Cell Signaling Technology (MA, USA) | - | 1:1000 | - |
| p-YAP (S127, rabbit mAb) | Cell Signaling Technology (MA, USA) | - | 1:1000 | - |
| MST1 | Cell Signaling Technology (MA, USA) | - | 1:1000 | - |
| p-MST1/2 (T183/180, rabbit mAb) | Cell Signaling Technology (MA, USA) | - | 1:1000 | - |
| LATS1 (rabbit mAb; C66B5) | Cell Signaling Technology (MA, USA) | - | 1:1000 | - |
| p-LATS (T1079, rabbit mAb; D57D3) | Cell Signaling Technology (MA, USA) | - | 1:1000 | - |
| GAPDH (rabbit mAb; 14C10) | Cell Signaling Technology (MA, USA) | - | 1:1000 | - |
| Histone H3 (rabbit mAb) | Santa cruz (CA,USA) | - | 1:1000 | - |

Abbreviations: mAb; monoclonal antibody, pAb; polyclonal antibody, p; phosphorylation, IHC; Immunohistochemistry, WB; Western blotting, IF; immunofluorescence

## Supplementary Table 2. List of gene signatures used in the present study

| Signature name | Description | Gene counts | Reference |
| --- | --- | --- | --- |
| Hoshida_subclass_S1 | Prognostic classifiers | 237 | 1 |
| Hoshida_subclass_S2 |  | 115 |  |
| Hoshida_subclass_S3 |  | 266 |  |
| Boyault_G1 | Prognostic classifiers | 116 | 2 |
| Boyault_G2 |  | 27 |  |
| Boyault_G3 |  | 189 |  |
| Boyault_G5 |  | 5 |  |
| Boyault_G6 |  | 66 |  |
| Yamashita_liver cancer stem_Up | Stemness-related signature | 48 | 3 |
| Yamashita_liver cancer stem_Down |  | 76 |  |

## Supplementary Table 3. List of primers and annealing conditions used for qRT-PCR and cloning

| Primer | Sequence (5’→3’) | Annealing |
| --- | --- | --- |
| *ESR1 F* | TCCAGCACCCTGAAGTCTCT | 62°C |
| *ESR1 R* | CGTAGACATGTCGTACTTCA |  |
| *MYBL2 F* | AGAAACGAGCCTGCCTTACA | 58°C |
| *MYBL2* R | ATCAAGTCCAGGGCTTCAGA |  |
| *CDC20* F | GAGGTGCAGCTATGGGATGT | 58°C |
| *CDC20* R | ACATCATGGTGGTGGATGTG |  |
| *TOP2A* F | GACGCTTCGTTATGGGAAGA | 58°C |
| *TOP2A R* | CAGAAAACGATGTCGCAGAA |  |
| *ACTB F* | TGGCACCCAGCACAATGAA | 56°C |
| *ACTB R* | CTAAGTCATAGTCCGCCTAGAAGCA |  |
| *infusion-ESR1 F* | CATAGAAGATTCTAGAATGACCATGACCCTCCACAC | 68°C |
| *infusion-ESR1 R* | CAGATCCTTGCGGCCGCTCAGACCGTGGCAGGGAAAC |  |

F, forward; R, reverse.

## Supplementary Table 4. Differentially expressed genes between ESR1-L and ESR-H HCCs (ESR1 signature)

| **Symbol** | **HCC with** | **HCC with** | **FC** | **Symbol** | **HCC with** | **HCC with** | **FC** |
| --- | --- | --- | --- | --- | --- | --- | --- |
|  | **ESR1-L (mean)** | **ESR1-H (mean)** |  |  | **ESR1-L (mean)** | **ESR1-H (mean)** |  |
| **Sig ESR1-L** |  |  |  | **Sig ESR1-H** |  |  |  |
| (n=482) |  |  |  | (n=785) |  |  |  |
| AFP | 4.36 | 1.66 | -2.70 | CYP2A6 | 3.46 | 7.29 | 3.83 |
| CD24 | 5.39 | 3.12 | -2.27 | CYP3A4 | 2.84 | 6.62 | 3.78 |
| S100P | 3.70 | 1.69 | -2.01 | CYP8B1 | 2.74 | 6.12 | 3.38 |
| SPP1 | 6.50 | 4.61 | -1.89 | SLC22A1 | 2.84 | 5.94 | 3.10 |
| EPCAM | 2.94 | 1.11 | -1.83 | SLC10A1 | 3.04 | 6.00 | 2.96 |
| GPC3 | 7.16 | 5.38 | -1.78 | TAT | 3.95 | 6.90 | 2.95 |
| CCL20 | 4.41 | 2.68 | -1.73 | HSD11B1 | 4.13 | 7.02 | 2.89 |
| SPINK1 | 5.95 | 4.30 | -1.65 | ADH1C | 4.83 | 7.61 | 2.78 |
| H19 | 5.01 | 3.37 | -1.64 | CYP2C8 | 4.05 | 6.73 | 2.68 |
| TESC | 3.39 | 1.78 | -1.61 | GLYAT | 1.94 | 4.61 | 2.66 |
| TMSB10 | 10.16 | 8.64 | -1.52 | LINC00844 | 1.32 | 3.94 | 2.62 |
| DLK1 | 1.74 | 0.25 | -1.50 | THRSP | 1.84 | 4.43 | 2.59 |
| GAL3ST1 | 2.51 | 1.02 | -1.49 | HPD | 5.90 | 8.41 | 2.51 |
| PYCR1 | 2.91 | 1.46 | -1.45 | ADH4 | 4.76 | 7.26 | 2.50 |
| DUSP9 | 2.79 | 1.34 | -1.44 | HSD17B13 | 2.16 | 4.64 | 2.48 |
| MYBL2 | 3.20 | 1.78 | -1.42 | TTC36 | 1.13 | 3.56 | 2.43 |
| EPS8L3 | 2.61 | 1.19 | -1.41 | AQP9 | 4.42 | 6.83 | 2.40 |
| BEX2 | 2.49 | 1.10 | -1.40 | LINC01018 | 1.31 | 3.66 | 2.35 |
| DKK1 | 1.91 | 0.52 | -1.39 | ADH1B | 5.30 | 7.65 | 2.34 |
| PEG10 | 3.11 | 1.74 | -1.37 | CYP1A2 | 0.81 | 3.14 | 2.33 |
| H2AFY2 | 3.24 | 1.88 | -1.36 | CYP2A7 | 0.97 | 3.23 | 2.26 |
| S100A14 | 3.68 | 2.33 | -1.35 | UGT2B15 | 4.72 | 6.93 | 2.21 |
| MDK | 6.02 | 4.66 | -1.35 | HAO2 | 2.05 | 4.25 | 2.20 |
| FXYD2 | 1.72 | 0.37 | -1.35 | SLC27A5 | 3.60 | 5.79 | 2.19 |
| UBE2C | 3.78 | 2.43 | -1.35 | UGT2B10 | 4.35 | 6.53 | 2.18 |
| ETV4 | 2.65 | 1.35 | -1.30 | RDH16 | 3.21 | 5.39 | 2.18 |
| CYBA | 4.10 | 2.82 | -1.28 | ACSM5 | 2.50 | 4.67 | 2.17 |
| SPINT1 | 2.48 | 1.22 | -1.26 | HSD17B6 | 5.49 | 7.64 | 2.15 |
| SPHK1 | 2.22 | 0.96 | -1.25 | CYP4F2 | 3.09 | 5.13 | 2.05 |
| PAFAH1B3 | 3.85 | 2.62 | -1.23 | LINC01554 | 1.32 | 3.37 | 2.05 |
| CA9 | 1.77 | 0.57 | -1.21 | GYS2 | 1.84 | 3.88 | 2.04 |
| ACSL4 | 4.99 | 3.80 | -1.19 | AKR1D1 | 2.18 | 4.21 | 2.03 |
| TRNP1 | 3.13 | 1.94 | -1.19 | LINC01485 | 2.82 | 4.85 | 2.03 |
| UPK3A | 1.99 | 0.82 | -1.17 | CYP7A1 | 2.34 | 4.36 | 2.02 |
| VIL1 | 3.03 | 1.87 | -1.16 | RTP3 | 2.52 | 4.53 | 2.01 |
| KCTD17 | 2.54 | 1.38 | -1.16 | UGT2B7 | 4.75 | 6.74 | 2.00 |
| BPIFB2 | 1.71 | 0.55 | -1.16 | PCK1 | 4.21 | 6.19 | 1.98 |
| HMGA1 | 5.40 | 4.24 | -1.15 | GSTA2 | 3.78 | 5.74 | 1.96 |
| PPAP2C | 1.87 | 0.72 | -1.15 | GLYATL1 | 2.25 | 4.21 | 1.95 |
| CDC20 | 3.35 | 2.20 | -1.15 | FETUB | 3.26 | 5.21 | 1.95 |
| PTP4A3 | 3.41 | 2.26 | -1.14 | C6 | 3.86 | 5.78 | 1.92 |
| LGALS3BP | 6.70 | 5.57 | -1.13 | F9 | 3.97 | 5.88 | 1.92 |
| NTS | 1.68 | 0.54 | -1.13 | UGT1A4 | 2.12 | 4.04 | 1.91 |
| PKM | 4.28 | 3.15 | -1.13 | SEC14L2 | 2.90 | 4.81 | 1.91 |
| B3GNT3 | 2.58 | 1.45 | -1.13 | FNDC5 | 1.09 | 3.00 | 1.91 |
| FGFR3 | 4.17 | 3.04 | -1.13 | AKR7A3 | 3.33 | 5.23 | 1.90 |
| UBD | 5.91 | 4.78 | -1.13 | SAA1 | 6.05 | 7.95 | 1.89 |
| VEGFB | 4.88 | 3.75 | -1.13 | FMO3 | 4.76 | 6.65 | 1.89 |
| MISP | 1.40 | 0.27 | -1.13 | ACSM2A | 2.93 | 4.81 | 1.88 |
| PRAME | 1.35 | 0.22 | -1.13 | BHMT | 4.05 | 5.93 | 1.88 |
| IGF2BP2 | 2.22 | 1.12 | -1.11 | APOF | 3.02 | 4.89 | 1.87 |
| DCDC2 | 2.52 | 1.42 | -1.10 | MFSD2A | 1.23 | 3.09 | 1.86 |
| ARID3A | 1.89 | 0.79 | -1.10 | HRG | 6.30 | 8.16 | 1.86 |
| MSI1 | 1.72 | 0.64 | -1.07 | CYP2B6 | 2.72 | 4.57 | 1.85 |
| GAS5 | 5.20 | 4.13 | -1.07 | HPR | 5.50 | 7.35 | 1.85 |
| MARCKSL1 | 5.50 | 4.43 | -1.07 | RP11-115C10.1 | 0.95 | 2.80 | 1.85 |
| SOX4 | 2.86 | 1.79 | -1.07 | ABCB4 | 2.52 | 4.35 | 1.84 |
| PTTG1 | 3.30 | 2.24 | -1.06 | MOGAT2 | 1.14 | 2.97 | 1.83 |
| MAPK13 | 1.85 | 0.79 | -1.06 | SULT2A1 | 5.98 | 7.78 | 1.81 |
| G6PD | 3.53 | 2.48 | -1.05 | C3P1 | 2.43 | 4.23 | 1.80 |
| KRT23 | 2.64 | 1.61 | -1.04 | CYP4A11 | 4.41 | 6.19 | 1.79 |
| CCNB1 | 3.40 | 2.37 | -1.03 | CES2 | 5.16 | 6.95 | 1.78 |
| MAL2 | 4.54 | 3.51 | -1.02 | ASPDH | 2.88 | 4.66 | 1.78 |
| SOX9 | 3.20 | 2.18 | -1.02 | SDS | 3.98 | 5.76 | 1.78 |
| KRT19 | 2.06 | 1.05 | -1.01 | SERPINC1 | 8.66 | 10.42 | 1.77 |
| QSOX1 | 3.13 | 2.12 | -1.00 | CYP2C9 | 4.80 | 6.55 | 1.75 |
| BIRC5 | 2.92 | 1.92 | -1.00 | GBA3 | 1.65 | 3.41 | 1.75 |
| LAD1 | 4.21 | 3.21 | -1.00 | PON1 | 4.89 | 6.64 | 1.75 |
| RPL8 | 9.64 | 8.64 | -1.00 | ACSM2B | 3.46 | 5.21 | 1.75 |
| RAB34 | 2.65 | 1.64 | -1.00 | C8A | 4.85 | 6.60 | 1.75 |
| HKDC1 | 3.29 | 2.30 | -1.00 | OTC | 4.11 | 5.85 | 1.74 |
| SLC29A4 | 2.01 | 1.02 | -0.99 | ETNPPL | 2.65 | 4.36 | 1.71 |
| PDE9A | 1.70 | 0.71 | -0.99 | SPP2 | 3.78 | 5.48 | 1.70 |
| PRR15L | 2.11 | 1.12 | -0.99 | ABCB11 | 1.31 | 3.00 | 1.69 |
| CLIC1 | 6.81 | 5.83 | -0.98 | SLC28A1 | 1.87 | 3.56 | 1.69 |
| SLC1A5 | 2.97 | 1.99 | -0.98 | MT1X | 3.79 | 5.47 | 1.69 |
| DBN1 | 2.69 | 1.72 | -0.97 | SLC46A3 | 2.08 | 3.76 | 1.67 |
| PHLDA2 | 2.93 | 1.97 | -0.96 | PDK4 | 3.26 | 4.93 | 1.67 |
| NGFRAP1 | 5.90 | 4.93 | -0.96 | CPS1 | 4.86 | 6.52 | 1.66 |
| CDHR2 | 2.15 | 1.19 | -0.96 | CCL16 | 3.84 | 5.49 | 1.65 |
| ITM2C | 4.55 | 3.59 | -0.96 | CFHR5 | 3.61 | 5.23 | 1.63 |
| PLP2 | 4.86 | 3.90 | -0.96 | ALDH1L1 | 3.45 | 5.07 | 1.63 |
| AURKB | 2.35 | 1.40 | -0.95 | CYP4A22 | 2.78 | 4.40 | 1.62 |
| GGT1 | 3.41 | 2.46 | -0.95 | ANXA10 | 1.62 | 3.24 | 1.61 |
| AGR2 | 1.37 | 0.42 | -0.94 | RP11-468N14.3 | 1.89 | 3.50 | 1.61 |
| NRSN2 | 2.42 | 1.48 | -0.94 | CCND2P1 | 1.66 | 3.26 | 1.61 |
| MMP9 | 2.63 | 1.69 | -0.94 | GBP7 | 1.90 | 3.50 | 1.60 |
| RAP1GAP | 3.23 | 2.30 | -0.94 | HP | 8.29 | 9.88 | 1.59 |
| IGF2BP1 | 1.41 | 0.48 | -0.94 | SAA2 | 3.25 | 4.84 | 1.59 |
| LPCAT1 | 2.97 | 2.05 | -0.93 | CFHR4 | 2.09 | 3.67 | 1.58 |
| S100A6 | 5.93 | 5.01 | -0.92 | TTR | 7.89 | 9.48 | 1.58 |
| TOP2A | 2.99 | 2.07 | -0.92 | EHHADH | 4.08 | 5.65 | 1.57 |
| AC009014.3 | 1.31 | 0.39 | -0.92 | HAO1 | 5.14 | 6.71 | 1.57 |
| NPTX2 | 1.41 | 0.50 | -0.91 | CYP2B7P | 1.66 | 3.22 | 1.57 |
| LAPTM4B | 5.19 | 4.28 | -0.91 | TSKU | 4.30 | 5.86 | 1.56 |
| SELM | 3.39 | 2.48 | -0.91 | TPRG1-AS1 | 1.38 | 2.93 | 1.55 |
| MFSD10 | 3.35 | 2.44 | -0.91 | DPYS | 4.37 | 5.92 | 1.54 |
| RPS12 | 9.85 | 8.95 | -0.90 | DAO | 2.42 | 3.97 | 1.54 |
| SNRPN | 4.04 | 3.14 | -0.90 | SLC25A47 | 3.08 | 4.61 | 1.53 |
| GNAZ | 2.06 | 1.17 | -0.90 | GPD1 | 2.66 | 4.19 | 1.53 |
| LRRC1 | 1.86 | 0.97 | -0.89 | AR | 1.61 | 3.14 | 1.52 |
| NCK2 | 3.07 | 2.17 | -0.89 | GSTA1 | 7.13 | 8.64 | 1.51 |
| BAIAP2L2 | 2.57 | 1.68 | -0.89 | PLG | 6.05 | 7.55 | 1.50 |
| TRIM50 | 1.33 | 0.44 | -0.89 | APOA5 | 5.07 | 6.57 | 1.50 |
| SLC6A8 | 2.30 | 1.41 | -0.89 | AFM | 4.36 | 5.84 | 1.48 |
| FGFR4 | 5.55 | 4.66 | -0.88 | DMGDH | 2.79 | 4.26 | 1.47 |
| SEL1L3 | 2.38 | 1.49 | -0.88 | RP4-763G1.2 | 1.98 | 3.45 | 1.47 |
| DMKN | 1.18 | 0.30 | -0.88 | SRD5A2 | 0.86 | 2.33 | 1.47 |
| SRC | 2.69 | 1.81 | -0.88 | LINC01093 | 0.61 | 2.08 | 1.47 |
| NEURL3 | 1.66 | 0.79 | -0.88 | C9 | 3.51 | 4.97 | 1.46 |
| MARCKS | 4.57 | 3.70 | -0.88 | CFHR3 | 2.79 | 4.25 | 1.46 |
| NT5DC2 | 2.66 | 1.78 | -0.87 | DCXR | 6.73 | 8.18 | 1.45 |
| CTNND2 | 1.18 | 0.31 | -0.87 | CTH | 3.03 | 4.47 | 1.45 |
| IER3 | 4.41 | 3.54 | -0.87 | GPLD1 | 1.46 | 2.91 | 1.45 |
| SOAT2 | 1.96 | 1.09 | -0.87 | AKR1C6P | 1.27 | 2.71 | 1.45 |
| TEAD2 | 3.08 | 2.21 | -0.87 | AOX1 | 5.19 | 6.63 | 1.44 |
| SNHG6 | 5.47 | 4.62 | -0.86 | NR1I2 | 1.53 | 2.97 | 1.44 |
| DDR1 | 2.25 | 1.40 | -0.85 | ALDOB | 8.44 | 9.88 | 1.43 |
| TNFRSF21 | 3.27 | 2.41 | -0.85 | RP11-372E1.4 | 1.82 | 3.25 | 1.43 |
| CDK1 | 2.41 | 1.55 | -0.85 | SLC13A5 | 3.27 | 4.69 | 1.42 |
| AC016735.1 | 1.28 | 0.43 | -0.85 | SLC51A | 2.51 | 3.93 | 1.41 |
| S100A11 | 6.39 | 5.55 | -0.85 | PCK2 | 5.29 | 6.71 | 1.41 |
| CENPM | 2.34 | 1.50 | -0.85 | SAA2-SAA4 | 2.27 | 3.68 | 1.41 |
| SOX12 | 2.91 | 2.06 | -0.85 | NAT2 | 1.10 | 2.51 | 1.41 |
| HID1 | 1.88 | 1.03 | -0.84 | SLC16A2 | 2.88 | 4.29 | 1.40 |
| CCNB2 | 2.34 | 1.50 | -0.84 | TTPA | 3.13 | 4.52 | 1.39 |
| CTHRC1 | 2.10 | 1.26 | -0.84 | UPB1 | 3.40 | 4.79 | 1.39 |
| C19orf48 | 4.25 | 3.41 | -0.84 | DIO1 | 3.95 | 5.31 | 1.37 |
| TPX2 | 3.26 | 2.42 | -0.84 | CMBL | 4.37 | 5.73 | 1.36 |
| SLC39A4 | 1.95 | 1.11 | -0.84 | AZGP1 | 6.70 | 8.06 | 1.36 |
| ZFAS1 | 4.40 | 3.56 | -0.84 | PFKFB1 | 1.90 | 3.26 | 1.36 |
| GDF15 | 5.09 | 4.25 | -0.84 | SLC27A2 | 4.34 | 5.70 | 1.36 |
| LYPD1 | 1.64 | 0.81 | -0.84 | IGSF23 | 2.15 | 3.50 | 1.35 |
| KIF2C | 2.02 | 1.19 | -0.83 | ABAT | 3.62 | 4.97 | 1.35 |
| MMP11 | 2.06 | 1.23 | -0.83 | MASP2 | 4.02 | 5.36 | 1.35 |
| COLCA2 | 1.41 | 0.59 | -0.83 | CYP1A1 | 1.13 | 2.48 | 1.35 |
| FOXQ1 | 1.86 | 1.04 | -0.83 | A1BG | 3.24 | 4.59 | 1.34 |
| PCSK1N | 1.23 | 0.41 | -0.82 | ANO1 | 2.01 | 3.36 | 1.34 |
| TRIM71 | 1.00 | 0.19 | -0.82 | CYP39A1 | 1.09 | 2.44 | 1.34 |
| H2AFX | 4.29 | 3.48 | -0.81 | TDO2 | 3.41 | 4.74 | 1.33 |
| CKS2 | 5.00 | 4.19 | -0.81 | ETNK2 | 3.63 | 4.96 | 1.33 |
| KIFC1 | 2.55 | 1.74 | -0.81 | RBP5 | 4.76 | 6.09 | 1.32 |
| PITX1 | 1.27 | 0.46 | -0.81 | GNMT | 3.96 | 5.28 | 1.32 |
| CAPG | 3.57 | 2.76 | -0.81 | CDO1 | 4.96 | 6.28 | 1.32 |
| C10orf35 | 2.19 | 1.39 | -0.81 | SLC2A2 | 5.41 | 6.73 | 1.32 |
| RPLP0 | 8.41 | 7.60 | -0.81 | SLC22A7 | 4.47 | 5.79 | 1.32 |
| RPS21 | 8.32 | 7.52 | -0.81 | F13B | 4.28 | 5.60 | 1.31 |
| UBE2T | 3.35 | 2.54 | -0.80 | SLC1A1 | 2.02 | 3.34 | 1.31 |
| RPS18 | 9.30 | 8.49 | -0.80 | ACSL1 | 5.06 | 6.37 | 1.31 |
| RPS24 | 7.30 | 6.50 | -0.80 | NR1I3 | 2.82 | 4.13 | 1.31 |
| SPINT2 | 2.14 | 1.34 | -0.80 | CUX2 | 1.40 | 2.70 | 1.30 |
| CDKN3 | 2.62 | 1.81 | -0.80 | CPN2 | 5.03 | 6.33 | 1.30 |
| CKAP4 | 5.27 | 4.47 | -0.80 | RAMP1 | 4.91 | 6.21 | 1.30 |
| PROM1 | 1.56 | 0.76 | -0.80 | IFI27 | 3.79 | 5.09 | 1.30 |
| TMED3 | 1.61 | 0.82 | -0.80 | INSIG1 | 5.72 | 7.02 | 1.30 |
| ALDOA | 6.16 | 5.36 | -0.80 | LAMA5-AS1 | 1.36 | 2.66 | 1.30 |
| NDRG1 | 4.46 | 3.66 | -0.79 | GSTM1 | 1.34 | 2.63 | 1.29 |
| SLC44A3 | 2.79 | 1.99 | -0.79 | ACADL | 0.72 | 2.01 | 1.29 |
| FAM3B | 1.95 | 1.16 | -0.78 | HNF4A-AS1 | 1.61 | 2.89 | 1.29 |
| NREP | 2.48 | 1.70 | -0.78 | FMO4 | 2.35 | 3.63 | 1.28 |
| TFF3 | 2.30 | 1.52 | -0.78 | KCNJ8 | 2.89 | 4.16 | 1.27 |
| GAPDH | 9.60 | 8.82 | -0.78 | ABCG8 | 3.37 | 4.64 | 1.27 |
| HIST3H2A | 1.48 | 0.70 | -0.78 | OGDHL | 2.69 | 3.95 | 1.27 |
| RPS19 | 7.68 | 6.90 | -0.78 | SCP2 | 4.50 | 5.77 | 1.27 |
| SNORD104 | 4.75 | 3.98 | -0.77 | FGGY | 2.61 | 3.88 | 1.27 |
| RPS2 | 7.98 | 7.21 | -0.77 | LDHD | 3.98 | 5.25 | 1.26 |
| LTB | 2.74 | 1.96 | -0.77 | SLCO1B1 | 4.40 | 5.66 | 1.26 |
| TNNC1 | 1.35 | 0.58 | -0.77 | MTHFD1 | 3.56 | 4.82 | 1.26 |
| WNK2 | 1.02 | 0.25 | -0.77 | ALDH6A1 | 3.79 | 5.04 | 1.25 |
| TTLL4 | 2.13 | 1.36 | -0.77 | MYRIP | 1.36 | 2.59 | 1.24 |
| RP11-20I20.4 | 2.77 | 2.00 | -0.77 | AADAC | 5.97 | 7.21 | 1.24 |
| PLEKHB1 | 1.00 | 0.24 | -0.77 | CES1 | 7.96 | 9.19 | 1.23 |
| TMC6 | 1.85 | 1.09 | -0.76 | HFE2 | 5.05 | 6.28 | 1.23 |
| SPATC1L | 1.85 | 1.09 | -0.76 | CTC-505O3.2 | 1.85 | 3.08 | 1.23 |
| NXPH4 | 1.44 | 0.67 | -0.76 | UGT2B17 | 0.85 | 2.08 | 1.23 |
| WBP5 | 4.34 | 3.58 | -0.76 | AGXT | 6.57 | 7.79 | 1.23 |
| ASPHD1 | 1.61 | 0.86 | -0.76 | SLC38A4 | 4.71 | 5.93 | 1.22 |
| FKBP10 | 2.63 | 1.87 | -0.76 | INHBC | 3.21 | 4.43 | 1.22 |
| LOXL4 | 2.70 | 1.95 | -0.75 | AGXT2 | 2.19 | 3.42 | 1.22 |
| TROAP | 1.85 | 1.10 | -0.75 | GCGR | 1.67 | 2.89 | 1.22 |
| RPL7P9 | 3.49 | 2.74 | -0.75 | KNG1 | 7.96 | 9.18 | 1.21 |
| NPM3 | 3.99 | 3.24 | -0.75 | SAA4 | 5.24 | 6.46 | 1.21 |
| ADM2 | 2.36 | 1.61 | -0.75 | PHYHD1 | 1.66 | 2.87 | 1.21 |
| CDC42EP1 | 5.93 | 5.19 | -0.75 | SORD | 3.87 | 5.07 | 1.21 |
| HN1 | 4.01 | 3.26 | -0.75 | CYP4F11 | 3.61 | 4.81 | 1.20 |
| PYCARD | 3.16 | 2.42 | -0.75 | HPX | 7.76 | 8.96 | 1.20 |
| BLVRA | 3.69 | 2.94 | -0.75 | FBP1 | 5.67 | 6.86 | 1.19 |
| LRRC75A-AS1 | 5.27 | 4.52 | -0.75 | ADH1A | 5.85 | 7.04 | 1.19 |
| RPSA | 6.84 | 6.10 | -0.74 | MIR621 | 5.03 | 6.22 | 1.19 |
| TMC4 | 1.82 | 1.07 | -0.74 | FABP4 | 1.55 | 2.74 | 1.19 |
| MUC5B | 0.96 | 0.22 | -0.74 | HORMAD2-AS1 | 1.13 | 2.32 | 1.19 |
| ASNS | 1.71 | 0.97 | -0.74 | SLC6A1 | 3.49 | 4.68 | 1.19 |
| RPL13A | 9.28 | 8.54 | -0.74 | MAT1A | 6.57 | 7.76 | 1.18 |
| BLMH | 2.62 | 1.88 | -0.74 | SELENBP1 | 5.07 | 6.25 | 1.18 |
| SMOX | 2.34 | 1.60 | -0.74 | XDH | 2.20 | 3.38 | 1.18 |
| CTB-63M22.1 | 3.14 | 2.40 | -0.74 | PIPOX | 5.13 | 6.31 | 1.18 |
| SNRPB | 7.03 | 6.29 | -0.74 | GPT2 | 3.68 | 4.86 | 1.18 |
| HSPB1 | 8.42 | 7.69 | -0.73 | SHMT1 | 4.01 | 5.18 | 1.18 |
| PLK1 | 1.88 | 1.15 | -0.73 | ECHDC3 | 4.07 | 5.24 | 1.17 |
| RENBP | 2.51 | 1.78 | -0.73 | CD14 | 6.32 | 7.49 | 1.17 |
| RPS11 | 9.46 | 8.72 | -0.73 | HAAO | 4.51 | 5.68 | 1.17 |
| BMF | 2.03 | 1.30 | -0.73 | SEPP1 | 5.83 | 7.00 | 1.17 |
| RP11-466H18.1 | 4.25 | 3.52 | -0.73 | G6PC | 5.63 | 6.80 | 1.17 |
| PPP1R14BP3 | 4.48 | 3.75 | -0.73 | ALPL | 2.56 | 3.72 | 1.17 |
| KIF20A | 1.96 | 1.23 | -0.73 | F12 | 6.16 | 7.32 | 1.17 |
| ANXA13 | 2.46 | 1.74 | -0.73 | APOC3 | 10.45 | 11.61 | 1.16 |
| ISYNA1 | 2.75 | 2.02 | -0.73 | KLKB1 | 3.75 | 4.92 | 1.16 |
| CDCA7 | 1.08 | 0.36 | -0.73 | IL27 | 1.48 | 2.64 | 1.16 |
| RPS6 | 8.73 | 8.00 | -0.73 | C4BPA | 7.31 | 8.47 | 1.16 |
| TLCD1 | 3.49 | 2.76 | -0.73 | ECM2 | 1.62 | 2.78 | 1.16 |
| SULT1C2 | 1.57 | 0.85 | -0.72 | C8B | 5.64 | 6.80 | 1.15 |
| HJURP | 1.79 | 1.06 | -0.72 | ABCA6 | 1.62 | 2.77 | 1.15 |
| DEPDC1B | 1.41 | 0.69 | -0.72 | BAAT | 6.34 | 7.49 | 1.15 |
| RRM2 | 2.82 | 2.10 | -0.72 | MTND4P20 | 2.05 | 3.20 | 1.15 |
| SLC16A3 | 1.77 | 1.05 | -0.72 | KLF9 | 3.35 | 4.49 | 1.14 |
| DDX39A | 4.13 | 3.42 | -0.72 | MTTP | 3.81 | 4.94 | 1.14 |
| RPS27 | 9.29 | 8.58 | -0.72 | RNU1-70P | 5.79 | 6.93 | 1.13 |
| MEP1A | 1.20 | 0.48 | -0.72 | PAGE4 | 0.55 | 1.68 | 1.13 |
| KIF12 | 3.08 | 2.36 | -0.72 | SPARCL1 | 3.58 | 4.70 | 1.12 |
| YWHAZ | 5.45 | 4.73 | -0.72 | ADORA2BP1 | 0.59 | 1.70 | 1.11 |
| NEK2 | 1.97 | 1.25 | -0.72 | AL161668.5 | 0.84 | 1.95 | 1.11 |
| CDC6 | 1.97 | 1.25 | -0.72 | BDH1 | 3.25 | 4.36 | 1.11 |
| RECQL4 | 2.62 | 1.90 | -0.72 | ANG | 6.81 | 7.92 | 1.11 |
| MKI67 | 1.91 | 1.19 | -0.72 | MBL2 | 3.08 | 4.19 | 1.11 |
| CENPW | 3.12 | 2.41 | -0.71 | ALDH8A1 | 3.66 | 4.77 | 1.11 |
| FBL | 5.73 | 5.02 | -0.71 | CAT | 5.80 | 6.90 | 1.10 |
| TCEAL8 | 4.47 | 3.76 | -0.71 | ACOT12 | 2.69 | 3.79 | 1.10 |
| SNHG1 | 2.91 | 2.20 | -0.71 | GREM2 | 0.95 | 2.05 | 1.10 |
| SLC38A1 | 2.23 | 1.52 | -0.71 | BHMT2 | 5.34 | 6.44 | 1.10 |
| TACC3 | 2.53 | 1.82 | -0.71 | F11 | 3.19 | 4.29 | 1.10 |
| RP11-295G20.2 | 3.53 | 2.82 | -0.71 | GNE | 2.98 | 4.08 | 1.09 |
| RP11-543P15.1 | 4.77 | 4.07 | -0.70 | CYP4F3 | 4.01 | 5.10 | 1.09 |
| HAGLR | 1.10 | 0.39 | -0.70 | RP13-650J16.1 | 1.41 | 2.50 | 1.09 |
| C4orf48 | 1.90 | 1.20 | -0.70 | NECAB2 | 1.56 | 2.65 | 1.09 |
| MFI2 | 1.22 | 0.52 | -0.70 | SLC1A2 | 1.30 | 2.38 | 1.09 |
| PPP1R14B | 5.10 | 4.40 | -0.70 | BBOX1 | 1.25 | 2.33 | 1.08 |
| ASF1B | 2.37 | 1.67 | -0.70 | MME | 0.80 | 1.88 | 1.08 |
| RPL7 | 7.57 | 6.86 | -0.70 | NUGGC | 1.27 | 2.35 | 1.08 |
| RPL9 | 6.84 | 6.14 | -0.70 | GADD45A | 4.04 | 5.11 | 1.07 |
| CDCA5 | 2.18 | 1.48 | -0.70 | AASS | 1.21 | 2.28 | 1.07 |
| NUF2 | 1.68 | 0.98 | -0.70 | ABCG2 | 1.56 | 2.63 | 1.07 |
| RPL22L1 | 4.08 | 3.38 | -0.70 | FAM99A | 1.12 | 2.19 | 1.07 |
| CDT1 | 2.27 | 1.58 | -0.70 | GHR | 2.05 | 3.12 | 1.07 |
| BCAM | 5.12 | 4.42 | -0.70 | GADD45G | 4.34 | 5.41 | 1.07 |
| GMNN | 4.14 | 3.45 | -0.70 | SORD2P | 1.28 | 2.34 | 1.07 |
| TRIM31 | 1.78 | 1.09 | -0.69 | SERPINA11 | 5.01 | 6.07 | 1.06 |
| TMEM51 | 2.08 | 1.39 | -0.69 | RP11-238F2.1 | 0.54 | 1.60 | 1.06 |
| GRAMD1A | 3.22 | 2.52 | -0.69 | ALDH2 | 5.32 | 6.38 | 1.06 |
| RPS3 | 6.82 | 6.13 | -0.69 | TM6SF2 | 1.92 | 2.97 | 1.05 |
| NPM1 | 6.59 | 5.90 | -0.69 | ACMSD | 3.40 | 4.45 | 1.05 |
| SUSD4 | 1.89 | 1.20 | -0.69 | RCAN1 | 2.14 | 3.19 | 1.05 |
| RPL23A | 7.04 | 6.35 | -0.69 | HLF | 2.61 | 3.66 | 1.05 |
| CLGN | 2.13 | 1.44 | -0.69 | ACOX2 | 3.77 | 4.81 | 1.05 |
| ATP1A1 | 6.21 | 5.52 | -0.69 | EPHX2 | 3.73 | 4.77 | 1.04 |
| ITPKA | 2.27 | 1.58 | -0.68 | DEPDC7 | 2.14 | 3.18 | 1.04 |
| GPX7 | 2.28 | 1.59 | -0.68 | SLC25A15 | 3.14 | 4.17 | 1.03 |
| SSR2 | 5.57 | 4.88 | -0.68 | AC005336.4 | 1.37 | 2.40 | 1.03 |
| ARHGEF2 | 2.21 | 1.53 | -0.68 | FXYD1 | 1.84 | 2.86 | 1.03 |
| PWWP2B | 2.79 | 2.11 | -0.68 | TMEM56 | 3.02 | 4.04 | 1.02 |
| MCCD1 | 0.91 | 0.23 | -0.68 | MPDZ | 1.57 | 2.58 | 1.01 |
| KPNA2 | 4.43 | 3.75 | -0.68 | GATM | 6.05 | 7.07 | 1.01 |
| C2orf82 | 2.54 | 1.86 | -0.68 | CYB5A | 4.65 | 5.66 | 1.01 |
| ZWINT | 3.07 | 2.39 | -0.68 | LECT2 | 3.49 | 4.50 | 1.01 |
| SLC6A11 | 1.18 | 0.50 | -0.68 | C1S | 7.24 | 8.25 | 1.01 |
| SNHG3 | 2.09 | 1.41 | -0.68 | DAK | 3.22 | 4.23 | 1.01 |
| NUSAP1 | 3.20 | 2.52 | -0.68 | DHRS1 | 2.93 | 3.93 | 1.01 |
| CENPF | 1.73 | 1.05 | -0.68 | EFHD1 | 1.62 | 2.62 | 1.01 |
| CENPA | 1.51 | 0.84 | -0.67 | RP11-42O15.3 | 2.13 | 3.13 | 1.00 |
| RP11-329L6.2 | 2.36 | 1.69 | -0.67 | GFRA1 | 1.14 | 2.14 | 1.00 |
| PDX1 | 1.12 | 0.45 | -0.67 | CYP2C18 | 3.17 | 4.17 | 1.00 |
| BAMBI | 3.82 | 3.15 | -0.67 | RP11-290F5.1 | 0.88 | 1.88 | 1.00 |
| MAGED1 | 4.91 | 4.24 | -0.67 | C16orf45 | 1.61 | 2.61 | 1.00 |
| EPO | 1.24 | 0.57 | -0.67 | OASL | 1.91 | 2.91 | 1.00 |
| LINGO1 | 1.30 | 0.63 | -0.67 | SERPINA4 | 5.40 | 6.40 | 1.00 |
| RPLP2 | 8.30 | 7.64 | -0.67 | ACY3 | 3.05 | 4.05 | 1.00 |
| RPL36A | 3.86 | 3.19 | -0.67 | MFAP3L | 0.80 | 1.80 | 1.00 |
| RPS5 | 7.17 | 6.50 | -0.67 | BCHE | 2.44 | 3.43 | 0.99 |
| LPAR2 | 1.39 | 0.72 | -0.67 | PON3 | 4.02 | 5.01 | 0.99 |
| RANGRF | 2.48 | 1.81 | -0.67 | ALDH1A1 | 7.63 | 8.62 | 0.99 |
| MEG3 | 1.13 | 0.46 | -0.66 | CDHR5 | 4.80 | 5.79 | 0.99 |
| ZIC2 | 1.46 | 0.80 | -0.66 | CPED1 | 0.65 | 1.64 | 0.99 |
| FBLN1 | 2.52 | 1.86 | -0.66 | C7 | 2.03 | 3.02 | 0.99 |
| FAM19A5 | 1.22 | 0.56 | -0.66 | SLCO2B1 | 3.44 | 4.43 | 0.98 |
| PEG3 | 1.11 | 0.45 | -0.66 | ECHDC2 | 3.16 | 4.15 | 0.98 |
| PFKP | 1.95 | 1.29 | -0.66 | COL5A3 | 2.23 | 3.21 | 0.98 |
| PNMA3 | 1.06 | 0.40 | -0.66 | OSGIN1 | 4.83 | 5.81 | 0.98 |
| SNRPD2 | 6.26 | 5.60 | -0.65 | ACAA2 | 4.91 | 5.89 | 0.98 |
| TMC5 | 1.08 | 0.43 | -0.65 | ALAS1 | 5.70 | 6.67 | 0.97 |
| H2AFZ | 5.61 | 4.95 | -0.65 | IGFBP2 | 5.00 | 5.97 | 0.97 |
| UNC5CL | 3.11 | 2.46 | -0.65 | ITIH1 | 7.32 | 8.29 | 0.97 |
| PTK7 | 1.31 | 0.66 | -0.65 | CYP2J2 | 3.68 | 4.65 | 0.97 |
| BAK1 | 3.15 | 2.50 | -0.65 | HAGH | 4.01 | 4.98 | 0.97 |
| IKBKE | 1.64 | 0.99 | -0.65 | DPP4 | 3.30 | 4.27 | 0.97 |
| TMEM132A | 1.43 | 0.78 | -0.65 | DNASE1L3 | 1.54 | 2.50 | 0.96 |
| NOP56 | 3.94 | 3.29 | -0.65 | AGMO | 3.22 | 4.18 | 0.96 |
| NRM | 2.97 | 2.32 | -0.65 | ANGPTL3 | 5.54 | 6.50 | 0.96 |
| RPOM1 | 6.40 | 5.75 | -0.65 | CES3 | 2.00 | 2.96 | 0.96 |
| RAB3D | 1.35 | 0.71 | -0.65 | MT2A | 6.49 | 7.45 | 0.96 |
| FOXJ1 | 0.85 | 0.20 | -0.65 | ACAT1 | 4.86 | 5.82 | 0.96 |
| C19orf33 | 1.30 | 0.65 | -0.65 | C1R | 6.63 | 7.58 | 0.96 |
| NMB | 2.41 | 1.76 | -0.65 | UGT1A2P | 0.55 | 1.51 | 0.95 |
| LMNB1 | 2.96 | 2.31 | -0.64 | ADH6 | 4.47 | 5.43 | 0.95 |
| SNHG7 | 2.91 | 2.26 | -0.64 | ANGPTL4 | 4.47 | 5.42 | 0.95 |
| CXCL5 | 0.96 | 0.32 | -0.64 | CGNL1 | 2.71 | 3.66 | 0.95 |
| SNAP25 | 1.18 | 0.54 | -0.64 | CTC-490E21.11 | 0.68 | 1.63 | 0.95 |
| RPL39L | 2.46 | 1.81 | -0.64 | CTSO | 3.57 | 4.51 | 0.95 |
| TAX1BP3 | 3.37 | 2.73 | -0.64 | UGT1A1 | 2.60 | 3.54 | 0.94 |
| DQX1 | 0.76 | 0.12 | -0.64 | PAH | 5.60 | 6.54 | 0.94 |
| CCT3 | 6.67 | 6.03 | -0.64 | KRT17P8 | 1.88 | 2.82 | 0.94 |
| RPL38 | 6.57 | 5.93 | -0.64 | ACSM1 | 2.24 | 3.18 | 0.94 |
| RPS8 | 8.04 | 7.40 | -0.64 | UBXN10 | 0.83 | 1.77 | 0.94 |
| RPS4X | 8.14 | 7.50 | -0.64 | PGRMC1 | 6.85 | 7.79 | 0.94 |
| UBE2S | 2.78 | 2.14 | -0.64 | SULT1B1 | 0.86 | 1.80 | 0.94 |
| CNOT11 | 4.32 | 3.68 | -0.64 | IFIT1 | 2.29 | 3.23 | 0.94 |
| RPS16 | 8.08 | 7.44 | -0.64 | HRSP12 | 6.37 | 7.31 | 0.94 |
| ACTG1 | 9.14 | 8.50 | -0.64 | SRD5A1 | 2.00 | 2.93 | 0.93 |
| PKN1 | 4.73 | 4.09 | -0.63 | ABCB1 | 2.45 | 3.38 | 0.93 |
| FKBP11 | 3.72 | 3.08 | -0.63 | ALAD | 4.50 | 5.43 | 0.93 |
| P3H4 | 2.27 | 1.63 | -0.63 | ADHFE1 | 2.38 | 3.31 | 0.93 |
| RPL35 | 8.00 | 7.36 | -0.63 | MYO1B | 3.88 | 4.81 | 0.93 |
| PSPH | 3.30 | 2.67 | -0.63 | EPHX1 | 8.98 | 9.92 | 0.93 |
| ADRA2C | 1.56 | 0.93 | -0.63 | GRHPR | 4.61 | 5.54 | 0.93 |
| ELF3 | 3.70 | 3.06 | -0.63 | LCAT | 3.04 | 3.97 | 0.93 |
| NAP1L1 | 3.65 | 3.01 | -0.63 | DHTKD1 | 4.03 | 4.96 | 0.93 |
| SERPINH1 | 4.47 | 3.84 | -0.63 | ABCC9 | 0.88 | 1.80 | 0.93 |
| RPL37A | 6.51 | 5.87 | -0.63 | FAM134B | 0.99 | 1.91 | 0.92 |
| NCAPG | 1.68 | 1.05 | -0.63 | SARDH | 3.49 | 4.41 | 0.92 |
| MMD | 2.77 | 2.14 | -0.63 | G0S2 | 4.89 | 5.81 | 0.92 |
| FXYD3 | 1.09 | 0.46 | -0.63 | 44078 | 1.49 | 2.40 | 0.92 |
| CHD3 | 2.11 | 1.48 | -0.63 | PTGR1 | 5.06 | 5.98 | 0.92 |
| HOMER3 | 2.22 | 1.59 | -0.63 | SLC17A4 | 2.69 | 3.61 | 0.92 |
| TSPAN15 | 2.69 | 2.06 | -0.63 | SERPINA10 | 4.58 | 5.49 | 0.91 |
| SLC52A2 | 3.52 | 2.90 | -0.62 | AC004862.6 | 0.57 | 1.48 | 0.91 |
| B4GALNT4 | 0.76 | 0.14 | -0.62 | KHK | 5.04 | 5.95 | 0.91 |
| FOXM1 | 2.16 | 1.54 | -0.62 | GSTZ1 | 1.67 | 2.58 | 0.91 |
| HNF1B | 2.18 | 1.55 | -0.62 | RGN | 4.70 | 5.61 | 0.91 |
| IGSF1 | 1.03 | 0.41 | -0.62 | MGST1 | 5.41 | 6.31 | 0.90 |
| PABPC1 | 7.58 | 6.96 | -0.62 | SYBU | 2.18 | 3.08 | 0.90 |
| KIAA0101 | 1.89 | 1.27 | -0.62 | METTL7A | 6.24 | 7.14 | 0.90 |
| RPL27 | 7.97 | 7.35 | -0.62 | CTSF | 4.64 | 5.54 | 0.90 |
| SLC34A2 | 0.87 | 0.26 | -0.62 | GSTA7P | 1.42 | 2.32 | 0.90 |
| RPL7A | 8.45 | 7.84 | -0.62 | RBP4 | 10.62 | 11.52 | 0.90 |
| RPL18A | 6.19 | 5.57 | -0.62 | SLC25A25 | 3.15 | 4.05 | 0.90 |
| PKIB | 1.49 | 0.87 | -0.62 | FBXO2 | 2.96 | 3.86 | 0.90 |
| SAPCD2 | 1.02 | 0.40 | -0.62 | ABCG5 | 2.84 | 3.73 | 0.89 |
| PNMA1 | 2.68 | 2.06 | -0.62 | RP11-132A1.6 | 1.06 | 1.95 | 0.89 |
| CDKN1C | 1.68 | 1.07 | -0.61 | ALDH5A1 | 3.77 | 4.66 | 0.89 |
| SLC1A7 | 1.08 | 0.47 | -0.61 | CTD-3098H1.2 | 1.08 | 1.97 | 0.88 |
| FLVCR1 | 1.92 | 1.30 | -0.61 | PC | 4.46 | 5.34 | 0.88 |
| RP11-465N4.4 | 1.74 | 1.13 | -0.61 | TMEM82 | 2.10 | 2.99 | 0.88 |
| LINC00152 | 2.32 | 1.71 | -0.61 | RP4-580N22.2 | 0.54 | 1.42 | 0.88 |
| TPM4 | 4.26 | 3.65 | -0.61 | CPB2 | 7.19 | 8.07 | 0.88 |
| RPL19 | 8.75 | 8.14 | -0.61 | PKLR | 4.59 | 5.47 | 0.88 |
| KIF4A | 1.84 | 1.23 | -0.61 | PEX11G | 1.95 | 2.83 | 0.88 |
| RPL32 | 7.49 | 6.88 | -0.61 | CYP27A1 | 6.54 | 7.42 | 0.88 |
| MCM3 | 4.46 | 3.85 | -0.61 | RORC | 3.52 | 4.40 | 0.87 |
| ESRP1 | 0.83 | 0.22 | -0.61 | ACSL5 | 3.90 | 4.77 | 0.87 |
| RPL35A | 6.86 | 6.25 | -0.61 | FCN2 | 0.44 | 1.31 | 0.87 |
| RPL18 | 7.09 | 6.49 | -0.61 | RP11-1259L22.2 | 0.49 | 1.36 | 0.87 |
| TYMS | 3.15 | 2.55 | -0.61 | HGD | 5.90 | 6.77 | 0.87 |
| ARL2 | 3.43 | 2.82 | -0.61 | PROZ | 2.65 | 3.52 | 0.87 |
| SNRPE | 5.43 | 4.82 | -0.61 | GCKR | 3.13 | 4.00 | 0.87 |
| CD7 | 1.84 | 1.24 | -0.61 | ACBD4 | 3.14 | 4.00 | 0.87 |
| RPS27A | 7.05 | 6.44 | -0.60 | UGT1A3 | 0.62 | 1.49 | 0.87 |
| LINC00665 | 1.20 | 0.60 | -0.60 | ACADM | 3.67 | 4.54 | 0.87 |
| LIMD2 | 2.06 | 1.46 | -0.60 | TMEM47 | 1.58 | 2.44 | 0.87 |
| FBLIM1 | 2.70 | 2.10 | -0.60 | TFR2 | 6.19 | 7.05 | 0.86 |
| TRIB3 | 4.57 | 3.97 | -0.60 | ST3GAL6 | 1.58 | 2.44 | 0.86 |
| CDCA8 | 2.19 | 1.59 | -0.60 | GCDH | 3.26 | 4.12 | 0.86 |
| MYRF | 2.73 | 2.13 | -0.60 | PXMP2 | 4.71 | 5.57 | 0.86 |
| NME1 | 4.23 | 3.63 | -0.60 | DBNDD1 | 2.13 | 2.99 | 0.86 |
| RP11-834C11.4 | 1.29 | 0.69 | -0.60 | PBLD | 3.27 | 4.12 | 0.86 |
| TRIM47 | 3.42 | 2.82 | -0.60 | RP11-328K4.1 | 1.27 | 2.12 | 0.86 |
| ZNF581 | 3.20 | 2.60 | -0.60 | SLC47A1 | 3.28 | 4.14 | 0.86 |
| COL9A2 | 1.01 | 0.42 | -0.60 | BANF1P2 | 1.76 | 2.62 | 0.86 |
| UCK2 | 2.67 | 2.08 | -0.60 | GPT | 4.19 | 5.04 | 0.85 |
| IMPDH1 | 2.21 | 1.61 | -0.60 | SERPING1 | 8.49 | 9.34 | 0.85 |
| SALL2 | 1.00 | 0.41 | -0.60 | ENPEP | 1.90 | 2.76 | 0.85 |
| BICC1 | 1.77 | 1.17 | -0.60 | RP11-119D9.1 | 0.53 | 1.38 | 0.85 |
| GTSE1 | 1.29 | 0.70 | -0.60 | AADAT | 1.03 | 1.88 | 0.85 |
| ATF4 | 6.86 | 6.27 | -0.59 | ETFDH | 2.93 | 3.79 | 0.85 |
| RP11-532F12.5 | 0.94 | 0.35 | -0.59 | PINK1 | 2.34 | 3.19 | 0.85 |
| MCM2 | 2.94 | 2.34 | -0.59 | HMGCS2 | 7.67 | 8.52 | 0.85 |
| RCC2 | 3.80 | 3.20 | -0.59 | ADI1 | 5.40 | 6.25 | 0.85 |
| NAT14 | 2.11 | 1.52 | -0.59 | RHOB | 6.85 | 7.70 | 0.85 |
| SEPW1 | 3.74 | 3.15 | -0.59 | RNASE4 | 2.22 | 3.07 | 0.85 |
| AURKA | 2.92 | 2.32 | -0.59 | ABCA8 | 0.88 | 1.73 | 0.85 |
| GTF3C6 | 5.13 | 4.54 | -0.59 | CADM1 | 2.49 | 3.33 | 0.85 |
| DANCR | 3.53 | 2.94 | -0.59 | RP11-434D9.1 | 0.36 | 1.21 | 0.85 |
| SCAMP5 | 2.15 | 1.56 | -0.59 | BOK | 4.36 | 5.20 | 0.85 |
| RPL23 | 6.55 | 5.96 | -0.59 | STEAP3 | 3.63 | 4.47 | 0.84 |
| TRIP13 | 1.45 | 0.86 | -0.59 | COPZ2 | 2.77 | 3.61 | 0.84 |
| RPL4 | 7.55 | 6.96 | -0.59 | MACROD1 | 2.90 | 3.75 | 0.84 |
| TGFB1 | 3.40 | 2.82 | -0.59 | PNPO | 3.72 | 4.56 | 0.84 |
| RP11-923I11.6 | 1.40 | 0.81 | -0.59 | TSC22D3 | 3.37 | 4.21 | 0.84 |
| RPL37 | 6.31 | 5.72 | -0.59 | ESR1 | 0.10 | 0.94 | 0.84 |
| ALYREF | 4.87 | 4.28 | -0.59 | SLC6A12 | 2.32 | 3.16 | 0.84 |
| GNB2L1 | 7.17 | 6.59 | -0.59 | SORBS2 | 1.99 | 2.83 | 0.84 |
| RPL28 | 6.48 | 5.89 | -0.58 | AVPR1A | 0.78 | 1.61 | 0.84 |
| RPL27A | 6.40 | 5.82 | -0.58 | RP11-813N20.1 | 0.85 | 1.69 | 0.84 |
| SMIM6 | 1.81 | 1.22 | -0.58 | RP11-21L23.2 | 1.05 | 1.89 | 0.83 |
| LMNB2 | 2.68 | 2.09 | -0.58 | MYLK | 1.52 | 2.35 | 0.83 |
| KCNF1 | 0.76 | 0.18 | -0.58 | HIBADH | 4.75 | 5.59 | 0.83 |
| LGALS2 | 1.94 | 1.35 | -0.58 | ELFN1 | 1.42 | 2.26 | 0.83 |
| NAALADL1 | 1.13 | 0.55 | -0.58 | DSG1 | 0.60 | 1.43 | 0.83 |
| TYRO3 | 1.05 | 0.46 | -0.58 | RP11-15I11.3 | 1.61 | 2.44 | 0.83 |
| SLC7A1 | 1.27 | 0.69 | -0.58 | SLC41A2 | 2.62 | 3.45 | 0.83 |
| CEP55 | 1.27 | 0.69 | -0.58 | PLGLA | 0.76 | 1.59 | 0.83 |
| RAN | 5.08 | 4.50 | -0.58 | LINC01151 | 0.97 | 1.80 | 0.83 |
| MPZL1 | 3.57 | 2.99 | -0.58 | NAMPT | 3.28 | 4.11 | 0.83 |
| CLSTN1 | 3.48 | 2.90 | -0.58 | RP11-622A1.2 | 1.56 | 2.39 | 0.83 |
| LZTS2 | 2.74 | 2.16 | -0.58 | SC5D | 3.55 | 4.38 | 0.83 |
| RPL5 | 7.78 | 7.20 | -0.58 | NDRG2 | 3.88 | 4.70 | 0.82 |
| C6orf48 | 5.20 | 4.62 | -0.58 | RP4-798A17.5 | 1.35 | 2.17 | 0.82 |
| ITGB4 | 1.79 | 1.21 | -0.58 | GCK | 0.36 | 1.18 | 0.82 |
| RPL6 | 6.94 | 6.36 | -0.58 | LIPG | 1.29 | 2.11 | 0.82 |
| SSTR5-AS1 | 0.81 | 0.23 | -0.58 | CYP4V2 | 2.59 | 3.41 | 0.82 |
| EGFR-AS1 | 0.81 | 0.23 | -0.58 | MASP1 | 2.45 | 3.26 | 0.81 |
| TRIM28 | 5.70 | 5.12 | -0.58 | ATP11C | 1.51 | 2.32 | 0.81 |
| DLGAP5 | 1.51 | 0.93 | -0.58 | RP11-513G11.3 | 0.61 | 1.42 | 0.81 |
| CACYBP | 3.88 | 3.30 | -0.58 | ACSS3 | 1.89 | 2.70 | 0.81 |
| ELOVL7 | 1.37 | 0.79 | -0.58 | RAMP3 | 2.53 | 3.34 | 0.81 |
| RPS10 | 6.40 | 5.82 | -0.58 | CP | 5.56 | 6.37 | 0.81 |
| EEF1B2 | 7.06 | 6.48 | -0.57 | FOLH1 | 1.27 | 2.08 | 0.81 |
| SKA1 | 1.54 | 0.97 | -0.57 | MIR568 | 2.29 | 3.09 | 0.81 |
| C15orf39 | 2.38 | 1.81 | -0.57 | SMIM14 | 4.10 | 4.90 | 0.81 |
| SPC25 | 1.59 | 1.02 | -0.57 | SORL1 | 1.78 | 2.58 | 0.80 |
| RPS20 | 8.13 | 7.55 | -0.57 | SLC17A2 | 2.89 | 3.69 | 0.80 |
| YBX3 | 2.15 | 1.58 | -0.57 | LEPR | 2.60 | 3.40 | 0.80 |
| RPLP1 | 8.48 | 7.90 | -0.57 | MYCL | 1.56 | 2.36 | 0.80 |
| PRR11 | 1.43 | 0.86 | -0.57 | CSAD | 2.24 | 3.03 | 0.80 |
| PRC1 | 2.17 | 1.60 | -0.57 | NAGS | 2.72 | 3.51 | 0.80 |
| SKA3 | 1.40 | 0.83 | -0.57 | ACAA1 | 4.04 | 4.84 | 0.80 |
| TGIF2 | 2.86 | 2.29 | -0.57 | SLC4A4 | 1.28 | 2.08 | 0.79 |
| C6orf223 | 0.87 | 0.30 | -0.57 | CYP7B1 | 1.46 | 2.25 | 0.79 |
| CBX6 | 1.97 | 1.40 | -0.57 | ARRDC4 | 1.88 | 2.68 | 0.79 |
| STMN1 | 3.74 | 3.17 | -0.57 | USP30-AS1 | 1.90 | 2.69 | 0.79 |
| NDC80 | 1.76 | 1.20 | -0.57 | RP11-320N7.2 | 0.67 | 1.47 | 0.79 |
| RP11-488L18.10 | 2.29 | 1.72 | -0.57 | KAT2B | 2.14 | 2.93 | 0.79 |
| TEAD4 | 1.80 | 1.23 | -0.57 | FAAH | 2.99 | 3.78 | 0.79 |
| ATIC | 4.43 | 3.86 | -0.57 | LEAP2 | 4.34 | 5.13 | 0.79 |
| E2F1 | 3.02 | 2.45 | -0.57 | CA2 | 3.77 | 4.56 | 0.79 |
| SNRPD1 | 3.06 | 2.49 | -0.57 | SLC30A1 | 3.63 | 4.41 | 0.79 |
| MYL6B | 3.12 | 2.56 | -0.57 | CLEC3B | 2.23 | 3.02 | 0.79 |
| CMTM3 | 2.59 | 2.03 | -0.57 | RP11-96D1.6 | 0.86 | 1.64 | 0.79 |
| RP11-285F7.2 | 3.44 | 2.87 | -0.57 | ABCC6 | 3.52 | 4.30 | 0.79 |
| FAM222A | 2.37 | 1.80 | -0.56 | ZNF385B | 0.75 | 1.54 | 0.78 |
| C11orf84 | 2.16 | 1.59 | -0.56 | KB-68A7.1 | 1.21 | 1.99 | 0.78 |
| MAPRE1 | 4.36 | 3.80 | -0.56 | AZGP1P1 | 1.95 | 2.73 | 0.78 |
| IQGAP3 | 1.91 | 1.35 | -0.56 | CLDN14 | 2.34 | 3.12 | 0.78 |
| RPL10A | 8.16 | 7.60 | -0.56 | DHODH | 2.29 | 3.06 | 0.78 |
| KIF18B | 1.34 | 0.78 | -0.56 | TMPRSS6 | 4.68 | 5.46 | 0.78 |
| GPR35 | 0.99 | 0.43 | -0.56 | HMGCL | 4.07 | 4.84 | 0.78 |
| MCM4 | 3.06 | 2.50 | -0.56 | ATP6V0E2 | 4.06 | 4.84 | 0.78 |
| C12orf49 | 1.96 | 1.40 | -0.56 | CD81 | 5.38 | 6.15 | 0.77 |
| TMEM147 | 5.08 | 4.52 | -0.56 | RCL1 | 2.34 | 3.11 | 0.77 |
| IGSF3 | 1.59 | 1.03 | -0.56 | MSMO1 | 5.67 | 6.44 | 0.77 |
| BUB1B | 1.37 | 0.81 | -0.56 | PIK3R1 | 2.57 | 3.34 | 0.77 |
| SLC45A4 | 1.17 | 0.61 | -0.56 | SPDYC | 0.91 | 1.68 | 0.77 |
| PALM | 1.94 | 1.39 | -0.56 | AMACR | 2.14 | 2.91 | 0.77 |
| ST6GALNAC4 | 2.08 | 1.52 | -0.56 | TMEM27 | 1.03 | 1.80 | 0.77 |
| HSP90AB1 | 8.60 | 8.05 | -0.56 | GABARAPL1 | 3.70 | 4.47 | 0.77 |
| IFITM10 | 1.76 | 1.20 | -0.56 | PRKAG2-AS1 | 1.66 | 2.43 | 0.76 |
| GUCA2A | 0.79 | 0.24 | -0.55 | SPTBN2 | 1.39 | 2.15 | 0.76 |
| RPL24 | 7.39 | 6.83 | -0.55 | HSD17B4 | 4.62 | 5.38 | 0.76 |
| RFC4 | 2.75 | 2.19 | -0.55 | RUNDC3B | 0.76 | 1.53 | 0.76 |
| RPLP0P6 | 2.36 | 1.80 | -0.55 | MAN1C1 | 0.92 | 1.68 | 0.76 |
| IMPDH2 | 5.27 | 4.72 | -0.55 | FAS | 1.53 | 2.29 | 0.76 |
| RPL18AP3 | 3.51 | 2.95 | -0.55 | LONP2 | 3.17 | 3.93 | 0.76 |
| FAM60A | 1.66 | 1.11 | -0.55 | LPA | 1.39 | 2.14 | 0.76 |
| RP11-175B9.3 | 3.52 | 2.97 | -0.55 | CFH | 6.43 | 7.19 | 0.76 |
| MIF | 5.46 | 4.91 | -0.55 | ADRA1B | 0.93 | 1.69 | 0.76 |
| MTHFD1L | 1.91 | 1.36 | -0.55 | KDM8 | 1.56 | 2.32 | 0.76 |
| UAP1L1 | 1.31 | 0.76 | -0.55 | ENTPD5 | 3.54 | 4.30 | 0.75 |
| PKDCC | 2.50 | 1.95 | -0.55 | HADH | 4.58 | 5.34 | 0.75 |
| VAT1 | 5.49 | 4.94 | -0.55 | ADRB2 | 0.95 | 1.70 | 0.75 |
| CPD | 4.00 | 3.44 | -0.55 | CCDC170 | 0.59 | 1.34 | 0.75 |
| SH3BGRL3 | 5.74 | 5.19 | -0.55 | UPP2 | 0.73 | 1.48 | 0.75 |
| RP11-800A18.4 | 0.67 | 0.12 | -0.55 | IYD | 0.89 | 1.64 | 0.75 |
| GINS1 | 1.74 | 1.19 | -0.55 | OLFM2 | 4.04 | 4.79 | 0.75 |
| IFNGR2 | 4.23 | 3.68 | -0.55 | RDH5 | 1.14 | 1.89 | 0.75 |
| DDIT3 | 4.70 | 4.15 | -0.55 | FCN3 | 1.05 | 1.79 | 0.75 |
|  |  |  |  | FCAMR | 0.69 | 1.43 | 0.75 |
|  |  |  |  | HYAL1 | 4.62 | 5.37 | 0.74 |
|  |  |  |  | CBR1 | 5.85 | 6.59 | 0.74 |
|  |  |  |  | RP11-122K13.7 | 1.13 | 1.87 | 0.74 |
|  |  |  |  | CPT2 | 3.55 | 4.29 | 0.74 |
|  |  |  |  | ACOX1 | 4.14 | 4.88 | 0.74 |
|  |  |  |  | UGP2 | 4.73 | 5.47 | 0.74 |
|  |  |  |  | APOL6 | 2.88 | 3.62 | 0.74 |
|  |  |  |  | KLF15 | 4.27 | 5.01 | 0.74 |
|  |  |  |  | GPR125 | 1.98 | 2.71 | 0.73 |
|  |  |  |  | DNAJC25 | 2.51 | 3.24 | 0.73 |
|  |  |  |  | IVD | 4.03 | 4.76 | 0.73 |
|  |  |  |  | ABHD2 | 3.80 | 4.53 | 0.73 |
|  |  |  |  | IFIT2 | 2.08 | 2.81 | 0.73 |
|  |  |  |  | CYP2A13 | 0.39 | 1.12 | 0.73 |
|  |  |  |  | ACADSB | 4.32 | 5.05 | 0.73 |
|  |  |  |  | TIGD2 | 1.96 | 2.69 | 0.73 |
|  |  |  |  | ACKR2 | 0.88 | 1.61 | 0.73 |
|  |  |  |  | PGM1 | 4.73 | 5.45 | 0.73 |
|  |  |  |  | POR | 5.94 | 6.67 | 0.73 |
|  |  |  |  | CRYL1 | 5.25 | 5.98 | 0.73 |
|  |  |  |  | COL18A1 | 6.07 | 6.79 | 0.73 |
|  |  |  |  | BTD | 3.45 | 4.17 | 0.72 |
|  |  |  |  | ANXA6 | 4.70 | 5.42 | 0.72 |
|  |  |  |  | TMEM220 | 2.84 | 3.56 | 0.72 |
|  |  |  |  | HEPACAM | 0.38 | 1.11 | 0.72 |
|  |  |  |  | KCND3 | 0.69 | 1.41 | 0.72 |
|  |  |  |  | 43892 | 4.62 | 5.34 | 0.72 |
|  |  |  |  | SLC6A13 | 0.83 | 1.55 | 0.72 |
|  |  |  |  | C4B | 4.30 | 5.02 | 0.72 |
|  |  |  |  | RP11-1182P23.5 | 0.52 | 1.24 | 0.72 |
|  |  |  |  | GLYCTK | 5.04 | 5.76 | 0.72 |
|  |  |  |  | ACSM3 | 1.32 | 2.04 | 0.72 |
|  |  |  |  | ENPP7 | 1.89 | 2.61 | 0.72 |
|  |  |  |  | LINC01348 | 1.43 | 2.15 | 0.72 |
|  |  |  |  | NFIA | 2.13 | 2.85 | 0.72 |
|  |  |  |  | CRY2 | 3.14 | 3.86 | 0.72 |
|  |  |  |  | ABHD6 | 2.71 | 3.43 | 0.72 |
|  |  |  |  | AP006216.5 | 0.68 | 1.40 | 0.72 |
|  |  |  |  | LRRC3 | 1.79 | 2.50 | 0.71 |
|  |  |  |  | ZBTB16 | 0.69 | 1.41 | 0.71 |
|  |  |  |  | ACACB | 2.14 | 2.85 | 0.71 |
|  |  |  |  | LRCOL1 | 0.93 | 1.64 | 0.71 |
|  |  |  |  | CES4A | 0.57 | 1.28 | 0.71 |
|  |  |  |  | ZG16 | 1.30 | 2.01 | 0.71 |
|  |  |  |  | FUOM | 5.24 | 5.95 | 0.71 |
|  |  |  |  | AP000355.2 | 1.39 | 2.10 | 0.71 |
|  |  |  |  | LRRC37A7P | 0.50 | 1.20 | 0.71 |
|  |  |  |  | AMDHD1 | 3.68 | 4.38 | 0.71 |
|  |  |  |  | FOLH1B | 0.36 | 1.07 | 0.70 |
|  |  |  |  | CNDP1 | 0.29 | 0.99 | 0.70 |
|  |  |  |  | LINC00261 | 3.90 | 4.61 | 0.70 |
|  |  |  |  | MROH2A | 0.22 | 0.92 | 0.70 |
|  |  |  |  | TMOD1 | 1.17 | 1.88 | 0.70 |
|  |  |  |  | ESPN | 3.73 | 4.43 | 0.70 |
|  |  |  |  | H6PD | 3.97 | 4.67 | 0.70 |
|  |  |  |  | DHRS4-AS1 | 2.22 | 2.92 | 0.70 |
|  |  |  |  | SAR1B | 3.27 | 3.97 | 0.70 |
|  |  |  |  | NNT | 3.51 | 4.21 | 0.70 |
|  |  |  |  | PPAP2B | 3.58 | 4.28 | 0.70 |
|  |  |  |  | RP3-434P1.6 | 0.98 | 1.68 | 0.70 |
|  |  |  |  | RP4-631H13.6 | 1.00 | 1.69 | 0.70 |
|  |  |  |  | PPARGC1A | 1.70 | 2.39 | 0.70 |
|  |  |  |  | CDC14B | 1.46 | 2.15 | 0.69 |
|  |  |  |  | CFL2 | 2.82 | 3.52 | 0.69 |
|  |  |  |  | SMARCA2 | 2.52 | 3.22 | 0.69 |
|  |  |  |  | PRAMEF10 | 0.31 | 1.00 | 0.69 |
|  |  |  |  | ALDH7A1 | 3.95 | 4.64 | 0.69 |
|  |  |  |  | RP11-266L9.8 | 1.75 | 2.44 | 0.69 |
|  |  |  |  | RP5-849H19.3 | 1.62 | 2.31 | 0.69 |
|  |  |  |  | APOL1 | 5.21 | 5.91 | 0.69 |
|  |  |  |  | ARHGEF26 | 1.76 | 2.45 | 0.69 |
|  |  |  |  | SLC22A25 | 1.41 | 2.10 | 0.69 |
|  |  |  |  | PROS1 | 4.71 | 5.40 | 0.69 |
|  |  |  |  | HSD17B8 | 4.43 | 5.12 | 0.69 |
|  |  |  |  | HDAC6 | 2.61 | 3.30 | 0.69 |
|  |  |  |  | TM7SF2 | 4.85 | 5.54 | 0.69 |
|  |  |  |  | RP11-19D2.1 | 1.47 | 2.16 | 0.68 |
|  |  |  |  | QDPR | 4.54 | 5.22 | 0.68 |
|  |  |  |  | ITPR2 | 2.62 | 3.30 | 0.68 |
|  |  |  |  | BCKDHB | 2.38 | 3.06 | 0.68 |
|  |  |  |  | CTD-2012K14.8 | 1.71 | 2.39 | 0.68 |
|  |  |  |  | ASB13 | 3.53 | 4.21 | 0.68 |
|  |  |  |  | PCSK6 | 2.48 | 3.16 | 0.68 |
|  |  |  |  | TTPAL | 1.86 | 2.54 | 0.68 |
|  |  |  |  | CYP11A1 | 1.01 | 1.69 | 0.68 |
|  |  |  |  | MLIP | 0.94 | 1.62 | 0.68 |
|  |  |  |  | NFIC | 3.67 | 4.35 | 0.68 |
|  |  |  |  | GADD45B | 4.92 | 5.60 | 0.68 |
|  |  |  |  | FAXDC2 | 2.79 | 3.47 | 0.67 |
|  |  |  |  | DUSP1 | 5.84 | 6.51 | 0.67 |
|  |  |  |  | MAOA | 4.19 | 4.86 | 0.67 |
|  |  |  |  | PALMD | 1.92 | 2.60 | 0.67 |
|  |  |  |  | GPHN | 1.85 | 2.53 | 0.67 |
|  |  |  |  | CRHBP | 0.38 | 1.05 | 0.67 |
|  |  |  |  | NBPF13P | 0.43 | 1.10 | 0.67 |
|  |  |  |  | AGL | 1.76 | 2.43 | 0.67 |
|  |  |  |  | RP4-539M6.20 | 0.63 | 1.30 | 0.67 |
|  |  |  |  | MERTK | 1.68 | 2.34 | 0.67 |
|  |  |  |  | CHAD | 2.66 | 3.32 | 0.67 |
|  |  |  |  | ADRA1A | 0.38 | 1.05 | 0.67 |
|  |  |  |  | RP11-798K3.2 | 3.29 | 3.96 | 0.67 |
|  |  |  |  | AUTS2 | 1.14 | 1.80 | 0.67 |
|  |  |  |  | COBLL1 | 1.83 | 2.49 | 0.66 |
|  |  |  |  | ACOT1 | 2.11 | 2.77 | 0.66 |
|  |  |  |  | AJ006998.2 | 0.40 | 1.07 | 0.66 |
|  |  |  |  | SLC31A1 | 3.90 | 4.56 | 0.66 |
|  |  |  |  | SYTL4 | 1.26 | 1.92 | 0.66 |
|  |  |  |  | ZMYND12 | 0.64 | 1.30 | 0.66 |
|  |  |  |  | MAOB | 5.70 | 6.36 | 0.66 |
|  |  |  |  | IRF6 | 3.58 | 4.24 | 0.66 |
|  |  |  |  | SLC35D1 | 3.07 | 3.73 | 0.66 |
|  |  |  |  | HULC | 3.20 | 3.85 | 0.66 |
|  |  |  |  | LHPP | 2.96 | 3.62 | 0.66 |
|  |  |  |  | CFI | 5.83 | 6.49 | 0.66 |
|  |  |  |  | HOMER2 | 1.53 | 2.19 | 0.66 |
|  |  |  |  | RBL2 | 2.17 | 2.83 | 0.66 |
|  |  |  |  | SHD | 1.07 | 1.73 | 0.66 |
|  |  |  |  | PYGL | 4.29 | 4.95 | 0.66 |
|  |  |  |  | GLS2 | 0.61 | 1.27 | 0.65 |
|  |  |  |  | IFIT3 | 2.92 | 3.57 | 0.65 |
|  |  |  |  | PPARA | 2.38 | 3.03 | 0.65 |
|  |  |  |  | F11-AS1 | 1.18 | 1.83 | 0.65 |
|  |  |  |  | ABTB2 | 1.84 | 2.49 | 0.65 |
|  |  |  |  | CTC-537E7.3 | 0.33 | 0.98 | 0.65 |
|  |  |  |  | DCAF11 | 3.98 | 4.63 | 0.65 |
|  |  |  |  | PPP1R3B | 3.12 | 3.77 | 0.65 |
|  |  |  |  | BX842568.1 | 0.56 | 1.21 | 0.65 |
|  |  |  |  | C4A | 4.03 | 4.68 | 0.65 |
|  |  |  |  | PAPSS2 | 3.47 | 4.12 | 0.65 |
|  |  |  |  | TGFBR3 | 1.28 | 1.93 | 0.65 |
|  |  |  |  | NFIX | 2.88 | 3.52 | 0.65 |
|  |  |  |  | RP11-7M8.2 | 0.55 | 1.20 | 0.65 |
|  |  |  |  | CRAT | 4.81 | 5.45 | 0.65 |
|  |  |  |  | GNAO1 | 0.36 | 1.01 | 0.65 |
|  |  |  |  | EHBP1 | 2.38 | 3.03 | 0.65 |
|  |  |  |  | RP11-403I13.5 | 0.65 | 1.30 | 0.64 |
|  |  |  |  | F5 | 5.44 | 6.09 | 0.64 |
|  |  |  |  | MIR135A1 | 3.37 | 4.02 | 0.64 |
|  |  |  |  | DGAT2 | 3.58 | 4.22 | 0.64 |
|  |  |  |  | TTC9 | 1.56 | 2.20 | 0.64 |
|  |  |  |  | IQGAP2 | 3.70 | 4.34 | 0.64 |
|  |  |  |  | FBXO31 | 2.76 | 3.40 | 0.64 |
|  |  |  |  | USP2 | 1.10 | 1.73 | 0.64 |
|  |  |  |  | PPL | 1.46 | 2.09 | 0.64 |
|  |  |  |  | RP11-611O2.2 | 1.43 | 2.07 | 0.64 |
|  |  |  |  | RORA | 1.07 | 1.70 | 0.64 |
|  |  |  |  | C16orf58 | 3.06 | 3.69 | 0.64 |
|  |  |  |  | BPHL | 2.89 | 3.53 | 0.64 |
|  |  |  |  | CCDC71L | 2.06 | 2.70 | 0.64 |
|  |  |  |  | SLC25A42 | 3.48 | 4.12 | 0.64 |
|  |  |  |  | GBP1 | 2.76 | 3.40 | 0.64 |
|  |  |  |  | SALL1 | 2.61 | 3.24 | 0.63 |
|  |  |  |  | PRKAG2 | 1.63 | 2.26 | 0.63 |
|  |  |  |  | SIAE | 2.22 | 2.86 | 0.63 |
|  |  |  |  | ZFAND5 | 4.23 | 4.87 | 0.63 |
|  |  |  |  | FAM13A | 0.72 | 1.35 | 0.63 |
|  |  |  |  | APBA1 | 0.72 | 1.36 | 0.63 |
|  |  |  |  | NUDT7 | 2.35 | 2.99 | 0.63 |
|  |  |  |  | TMEM150C | 1.92 | 2.55 | 0.63 |
|  |  |  |  | CTD-2619J13.27 | 1.02 | 1.65 | 0.63 |
|  |  |  |  | PROL1 | 0.43 | 1.06 | 0.63 |
|  |  |  |  | MUT | 4.31 | 4.93 | 0.63 |
|  |  |  |  | N4BP2L1 | 1.83 | 2.46 | 0.63 |
|  |  |  |  | IFI44 | 2.43 | 3.05 | 0.63 |
|  |  |  |  | IFNLR1 | 0.91 | 1.53 | 0.62 |
|  |  |  |  | CYP3A43 | 0.60 | 1.23 | 0.62 |
|  |  |  |  | RNF144B | 1.60 | 2.22 | 0.62 |
|  |  |  |  | CPEB3 | 0.77 | 1.40 | 0.62 |
|  |  |  |  | AC009166.5 | 1.01 | 1.63 | 0.62 |
|  |  |  |  | ALDH4A1 | 5.57 | 6.20 | 0.62 |
|  |  |  |  | ALDH9A1 | 4.90 | 5.52 | 0.62 |
|  |  |  |  | GBE1 | 3.16 | 3.78 | 0.62 |
|  |  |  |  | PROC | 6.22 | 6.84 | 0.62 |
|  |  |  |  | SOCS2 | 1.14 | 1.76 | 0.62 |
|  |  |  |  | MIR1295A | 0.79 | 1.41 | 0.62 |
|  |  |  |  | RP11-635N19.1 | 1.48 | 2.10 | 0.62 |
|  |  |  |  | TMEM176B | 8.44 | 9.06 | 0.62 |
|  |  |  |  | NIPSNAP3A | 2.66 | 3.28 | 0.62 |
|  |  |  |  | PER1 | 2.76 | 3.38 | 0.62 |
|  |  |  |  | RP11-169K16.6 | 0.94 | 1.56 | 0.62 |
|  |  |  |  | SMOC1 | 3.73 | 4.34 | 0.62 |
|  |  |  |  | VSIG2 | 0.69 | 1.30 | 0.62 |
|  |  |  |  | FAM99B | 0.66 | 1.28 | 0.61 |
|  |  |  |  | ECI2 | 4.87 | 5.49 | 0.61 |
|  |  |  |  | MRC1 | 1.55 | 2.17 | 0.61 |
|  |  |  |  | EVA1A | 3.53 | 4.14 | 0.61 |
|  |  |  |  | TRIM22 | 1.64 | 2.25 | 0.61 |
|  |  |  |  | ARMC5 | 1.71 | 2.33 | 0.61 |
|  |  |  |  | ACE2 | 0.58 | 1.19 | 0.61 |
|  |  |  |  | LAMP2 | 5.36 | 5.98 | 0.61 |
|  |  |  |  | SLC2A10 | 2.54 | 3.15 | 0.61 |
|  |  |  |  | AOC4P | 1.29 | 1.90 | 0.61 |
|  |  |  |  | IGFBP4 | 8.01 | 8.62 | 0.61 |
|  |  |  |  | BLVRB | 6.05 | 6.66 | 0.61 |
|  |  |  |  | FZD4 | 2.02 | 2.63 | 0.61 |
|  |  |  |  | PITPNM2 | 1.57 | 2.17 | 0.61 |
|  |  |  |  | RP11-252E2.2 | 0.19 | 0.80 | 0.61 |
|  |  |  |  | KMO | 1.44 | 2.04 | 0.60 |
|  |  |  |  | KCNB1 | 0.36 | 0.96 | 0.60 |
|  |  |  |  | AC080008.1 | 0.65 | 1.25 | 0.60 |
|  |  |  |  | PANK1 | 2.23 | 2.83 | 0.60 |
|  |  |  |  | AC144652.1 | 0.83 | 1.43 | 0.60 |
|  |  |  |  | ADAMTSL4 | 2.06 | 2.66 | 0.60 |
|  |  |  |  | DBH | 0.82 | 1.42 | 0.60 |
|  |  |  |  | ARID3C | 1.15 | 1.75 | 0.60 |
|  |  |  |  | BCO2 | 0.41 | 1.01 | 0.60 |
|  |  |  |  | SOWAHB | 1.72 | 2.32 | 0.60 |
|  |  |  |  | SH3D19 | 2.68 | 3.28 | 0.60 |
|  |  |  |  | KLHL2 | 2.03 | 2.63 | 0.60 |
|  |  |  |  | EPB41L4B | 2.24 | 2.84 | 0.60 |
|  |  |  |  | TOB1 | 5.08 | 5.68 | 0.60 |
|  |  |  |  | RANBP10 | 2.08 | 2.67 | 0.60 |
|  |  |  |  | FAH | 4.11 | 4.71 | 0.60 |
|  |  |  |  | SLC2A12 | 0.64 | 1.24 | 0.60 |
|  |  |  |  | AC008592.4 | 0.43 | 1.03 | 0.59 |
|  |  |  |  | ACOT2 | 3.27 | 3.86 | 0.59 |
|  |  |  |  | OCIAD2 | 3.86 | 4.46 | 0.59 |
|  |  |  |  | MAP3K5 | 1.35 | 1.94 | 0.59 |
|  |  |  |  | TRIM35 | 1.89 | 2.48 | 0.59 |
|  |  |  |  | CD302 | 2.26 | 2.85 | 0.59 |
|  |  |  |  | NUDT16P1 | 1.32 | 1.91 | 0.59 |
|  |  |  |  | C1RL | 4.15 | 4.74 | 0.59 |
|  |  |  |  | HMGN5 | 1.01 | 1.60 | 0.59 |
|  |  |  |  | LIMS2 | 1.87 | 2.46 | 0.59 |
|  |  |  |  | CISH | 2.31 | 2.89 | 0.59 |
|  |  |  |  | ANGPTL6 | 0.84 | 1.43 | 0.59 |
|  |  |  |  | CA5A | 1.16 | 1.74 | 0.59 |
|  |  |  |  | LPIN2 | 4.09 | 4.68 | 0.59 |
|  |  |  |  | PEMT | 4.34 | 4.93 | 0.59 |
|  |  |  |  | FBLN5 | 1.80 | 2.39 | 0.59 |
|  |  |  |  | KCNJ4 | 0.79 | 1.37 | 0.59 |
|  |  |  |  | GOT2 | 5.51 | 6.10 | 0.59 |
|  |  |  |  | RP4-639F20.1 | 2.65 | 3.23 | 0.58 |
|  |  |  |  | CBR4 | 2.24 | 2.82 | 0.58 |
|  |  |  |  | IRS1 | 2.51 | 3.10 | 0.58 |
|  |  |  |  | CD1D | 1.18 | 1.76 | 0.58 |
|  |  |  |  | SDPR | 2.02 | 2.60 | 0.58 |
|  |  |  |  | CDC37L1 | 2.47 | 3.06 | 0.58 |
|  |  |  |  | OAF | 5.52 | 6.10 | 0.58 |
|  |  |  |  | HERC5 | 1.00 | 1.58 | 0.58 |
|  |  |  |  | CTD-3193O13.1 | 0.39 | 0.97 | 0.58 |
|  |  |  |  | RP11-553L6.5 | 3.47 | 4.05 | 0.58 |
|  |  |  |  | MNS1 | 0.80 | 1.38 | 0.58 |
|  |  |  |  | MLXIPL | 5.33 | 5.91 | 0.58 |
|  |  |  |  | RNF125 | 0.86 | 1.44 | 0.58 |
|  |  |  |  | RPL39P40 | 1.84 | 2.42 | 0.58 |
|  |  |  |  | NLRP6 | 0.56 | 1.14 | 0.58 |
|  |  |  |  | KDR | 1.85 | 2.43 | 0.58 |
|  |  |  |  | ARSD | 2.73 | 3.31 | 0.58 |
|  |  |  |  | ATF3 | 2.66 | 3.24 | 0.58 |
|  |  |  |  | VWF | 2.48 | 3.05 | 0.58 |
|  |  |  |  | SLCO2A1 | 1.20 | 1.77 | 0.58 |
|  |  |  |  | ACY1 | 2.19 | 2.77 | 0.58 |
|  |  |  |  | DEXI | 2.07 | 2.64 | 0.57 |
|  |  |  |  | SLC39A8 | 2.01 | 2.59 | 0.57 |
|  |  |  |  | ATOH8 | 1.04 | 1.61 | 0.57 |
|  |  |  |  | CHRNA4 | 0.51 | 1.08 | 0.57 |
|  |  |  |  | SLC16A13 | 3.23 | 3.80 | 0.57 |
|  |  |  |  | PECR | 4.40 | 4.97 | 0.57 |
|  |  |  |  | GSTK1 | 5.60 | 6.17 | 0.57 |
|  |  |  |  | TPRG1L | 4.63 | 5.20 | 0.57 |
|  |  |  |  | PQLC1 | 4.32 | 4.89 | 0.57 |
|  |  |  |  | SUCLG2 | 4.89 | 5.46 | 0.57 |
|  |  |  |  | FDX1 | 3.49 | 4.05 | 0.57 |
|  |  |  |  | RP5-966M1.6 | 2.06 | 2.63 | 0.57 |
|  |  |  |  | CD99L2 | 3.54 | 4.11 | 0.57 |
|  |  |  |  | SETD7 | 2.33 | 2.90 | 0.57 |
|  |  |  |  | SOD1 | 7.63 | 8.20 | 0.57 |
|  |  |  |  | OIT3 | 1.91 | 2.47 | 0.57 |
|  |  |  |  | TST | 6.90 | 7.47 | 0.57 |
|  |  |  |  | PIK3C2G | 0.83 | 1.40 | 0.56 |
|  |  |  |  | GMPR | 1.24 | 1.80 | 0.56 |
|  |  |  |  | PCCB | 3.50 | 4.06 | 0.56 |
|  |  |  |  | COLEC10 | 0.46 | 1.03 | 0.56 |
|  |  |  |  | APOL3 | 2.65 | 3.22 | 0.56 |
|  |  |  |  | TMEM25 | 0.77 | 1.33 | 0.56 |
|  |  |  |  | SLC38A2 | 4.41 | 4.97 | 0.56 |
|  |  |  |  | RAPGEF4 | 1.38 | 1.94 | 0.56 |
|  |  |  |  | DKFZp779M0652 | 1.78 | 2.35 | 0.56 |
|  |  |  |  | AADACP1 | 0.92 | 1.48 | 0.56 |
|  |  |  |  | DHX58 | 2.05 | 2.61 | 0.56 |
|  |  |  |  | CHPT1 | 3.93 | 4.49 | 0.56 |
|  |  |  |  | TPMT | 3.87 | 4.43 | 0.56 |
|  |  |  |  | CSRP2 | 1.84 | 2.40 | 0.56 |
|  |  |  |  | NAMPTP1 | 1.02 | 1.58 | 0.56 |
|  |  |  |  | PMEL | 0.91 | 1.47 | 0.56 |
|  |  |  |  | LINC00526 | 1.72 | 2.28 | 0.56 |
|  |  |  |  | DDX60 | 1.44 | 2.00 | 0.56 |
|  |  |  |  | ALDH1B1 | 4.71 | 5.27 | 0.56 |
|  |  |  |  | TRPM8 | 1.24 | 1.80 | 0.56 |
|  |  |  |  | FAM20A | 3.27 | 3.83 | 0.56 |
|  |  |  |  | STEAP4 | 0.63 | 1.19 | 0.56 |
|  |  |  |  | SMIM19 | 2.72 | 3.28 | 0.56 |
|  |  |  |  | PIGV | 2.36 | 2.91 | 0.55 |
|  |  |  |  | MAFB | 2.77 | 3.32 | 0.55 |
|  |  |  |  | SUOX | 2.93 | 3.49 | 0.55 |
|  |  |  |  | PHYH | 5.65 | 6.20 | 0.55 |
|  |  |  |  | FITM1 | 0.73 | 1.28 | 0.55 |
|  |  |  |  | NT5DC1 | 2.02 | 2.57 | 0.55 |
|  |  |  |  | BTNL9 | 0.85 | 1.40 | 0.55 |
|  |  |  |  | GCLC | 3.17 | 3.72 | 0.55 |
|  |  |  |  | DHRS3 | 5.93 | 6.48 | 0.55 |

Abbreviations: HCC, hepatocellular carcinoma; FC, fold change

## Supplementary Table 5. Comparison of the clinicopathological features between ESR1-H and ESR1-L HCCs in TCGA-LIHC

| **Clinicopathologic features** | **ESR1-H** | **ESR1-L** | ***p*-value** | |
| --- | --- | --- | --- | --- |
|  | **n=185** | **n=186** |  |  |
| Age (year, median, IQR) | 62 (20 - 90) | 57 (16 - 85) | | < 0.001 |
| Gender (male/female, %) | 140 (75.7%) / 45 (24.3%) | 110 (59.1%) / 76 (40.9%) | | 1 |
| Etiology (HBV/HCV/Alcohol/other, %) | 44 (44%) / 11 (11%) / 32 (32%) / 13 (13%) | 31 (30%) / 21 (20.4%) / 36 (35%) / 15 (14.6%) | | 0.13 |
| Albumin (median, IQR) | 4.7 (0.2 - 51) | 3.8 (0.4 - 6.9) | | 0.10 |
| Alpha-fetoprotein (median, IQR) | 767 (1.0 - 8.5x10^4^) | 28,399 (1.0 - 2x10^6^) | | < 0.001 |
| Tumor differentiation grade (G1/G2/G3/G4, %) | 39 (21.4%) / 98 (53.9%) / 43 (23.6%) / 2 (1.1%) | 16 (8.7%) / 79 (42.9%) / 79 (42.9%) / 10 (5.5%) | | < 0.001 |

Abbreviations: IQR, interquartile range; SD; standard deviation

## **Supplementary** Table 6. Univariate and multivariate Cox analysis of overall survival on HCC patients in TCGA-LIHC

| Variable |  | Univariate | | |  | Multivariate | | |
| --- | --- | --- | --- | --- | --- | --- | --- | --- |
|  | No. of patients (n=371) | HR | 95% CI | *p* value |  | HR | 95% CI | *p-*value |
| Age (>median) |  | 1.2 | 0.85-1.7 | 0.27 |  | - | - | - |
| high | 179 |  |  |  |  |  |  |  |
| low | 192 |  |  |  |  |  |  |  |
| Gender |  | 0.89 | 0.62-1.3 | 0.55 |  | - | - | - |
| Male | 250 |  |  |  |  |  |  |  |
| Female | 121 |  |  |  |  |  |  |  |
| Alpha-fetoprotein (>median) |  | 1.1 | 0.67-1.8 | 0.7 |  | - | - | - |
| high | 66 |  |  |  |  |  |  |  |
| low | 212 |  |  |  |  |  |  |  |
| Albumin (>median) |  | 0.72 | 0.47-1.1 | 0.13 |  | 0.72 | 0.47-1.11 | 0.135 |
| high | 160 |  |  |  |  |  |  |  |
| low | 137 |  |  |  |  |  |  |  |
| Tumor differentiation grade |  | 0.83 | 0.57-1.2 | 0.32 |  | - | - | - |
| G1-G2 | 232 |  |  |  |  |  |  |  |
| G3-G4 | 134 |  |  |  |  |  |  |  |
| ERα expression |  | 0.53 | 0.37-0.77 | **< 0.001** |  | 0.62 | 0.41-0.95 | **0.029** |
| Present | 185 |  |  |  |  |  |  |  |
| Absent | 186 |  |  |  |  |  |  |  |

Abbreviations: HR, hazard ratio; CI, confidence interval;

**References for Supplementary Tables**

1. Hoshida, Y. et al. Integrative transcriptome analysis reveals common molecular subclasses of human hepatocellular carcinoma. Cancer Res 69, 7385-7392, doi:10.1158/0008-5472.CAN-09-1089 (2009).

2. Boyault, S. et al. Transcriptome classification of HCC is related to gene alterations and to new therapeutic targets. Hepatology 45, 42-52, doi:10.1002/hep.21467 (2007).

3. Yamashita, T. et al. EpCAM-positive hepatocellular carcinoma cells are tumor-initiating cells with stem/progenitor cell features. Gastroenterology 136, 1012-1024, doi:10.1053/j.gastro.2008.12.004 (2009).

# Supplementary Figures

**
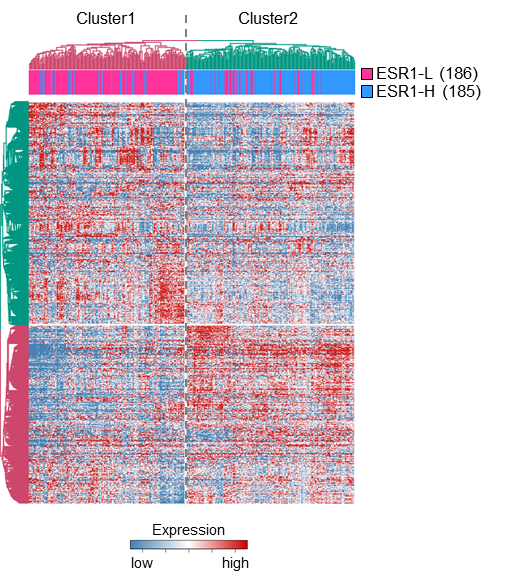
**

## Supplementary Fig. 1. Unsupervised clustering according to ESR1 expression status

A heatmap shows unsupervised clustering analysis using variably expressed genes (median absolute deviation, MAD > 0.5, n=7,094). ESR1-L (n = 186) and ESR1-H (n = 185) cases are indicated.


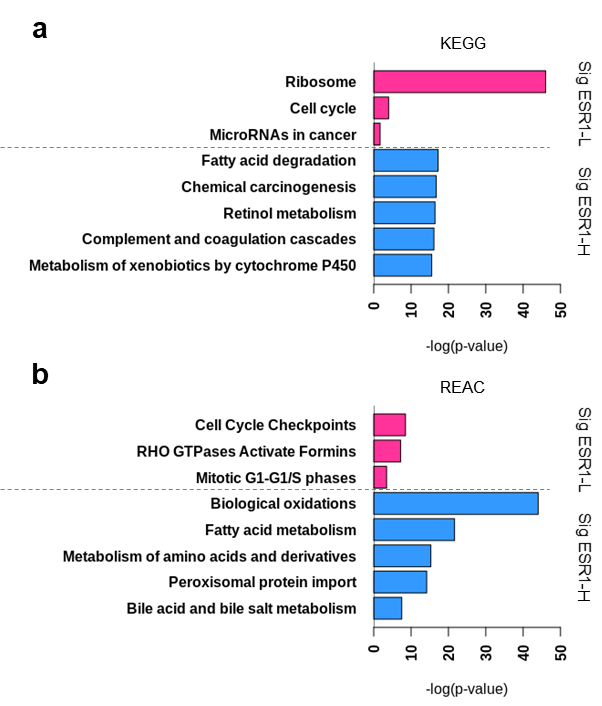


## Supplementary Fig. 2. Gene set analysis using ESR1 signatures in KEGG and REAC database

The gene signatures enriched in Sig ESR1-L and Sig ESR1-H are shown using KEGG (http://www.genome.jp/kegg/) (**a**) and REAC (http://www.reactome.org/) (**b**) databases, respectively. Plots represent log_10_ *p*-value rate range detected across gProfileR (ver.0.7.0) levels for top-ranked signatures using pink color for Sig ESR1-L and blue color for Sig ESR1-H.


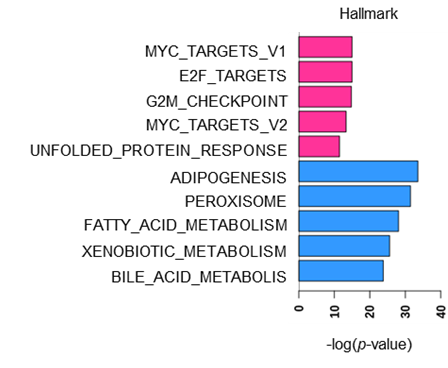


## Supplementary Fig. 3. Distribution of oncogenes and tumor suppressor genes in ESR1 signature

The gene signatures enriched in Sig ESR1-L and Sig ESR1-H are shown using Hallmark database (v7.0 MSigDB, <http://www.gsea-msigWdb.org/gsea/msigdb>). Plots represent log_10_ *p*-value rate range detected across differentially enrichment scores levels (ESR1-L vs. ESR1-H, Student’s t-test) for top-ranked signatures using pink color for Sig ESR1-L and blue color for Sig ESR1-H.

##
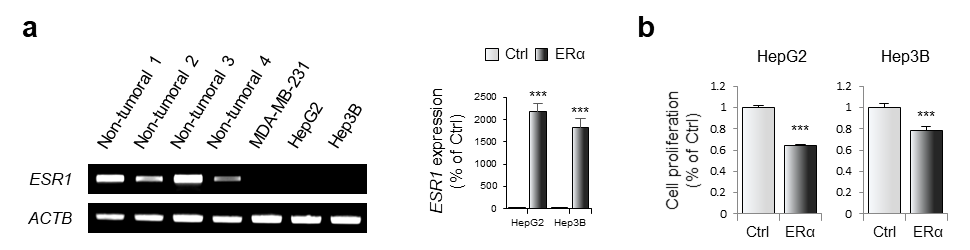


## Supplementary Fig. 4. Establishment of ERα (+) HCC cell lines and evaluation of cell proliferation

(**a**) RT-PCR results show expression of ESR1 on Non-tumoral liver tissues (adjacent tissue from four HCC patients), MDA-MB-231 (breast cancer cell line) and HCC cell lines (e.g., HepG2 and Hep3B). Non-tumoral liver tissues and MDA-MB-231 were used for ERα (+) and ERα (-), respectively (*left*). HepG2 and Hep3B were transfected with control (Ctrl, pCDM-CMV-MCS-EF1-puro vector) or vector encoding ERα and were maintained in puromycin (0.4 and 1.0 µg/ml) for 4 weeks. The expression of *ESR1* was assessed by quantitative RT-PCR. *ACTB*, housekeeping gene, was used for normalization (*right*). (**b**) The cell proliferation was assessed using MTT assay in HepG2 and Hep3B. Statistical significance is indicated (Ctrl vs. ERα; ****p* < 0.001, Student’s T-test).
